# Nuclear Isoforms of Neurofibromin Are Required for Proper Spindle Organization and Chromosome Segregation

**DOI:** 10.3390/cells9112348

**Published:** 2020-10-23

**Authors:** Charoula Peta, Emmanouella Tsirimonaki, Dimitris Samouil, Kyriaki Georgiadou, Dimitra Mangoura

**Affiliations:** Basic Research Center, Biomedical Research Foundation of the Academy of Athens, 4 Soranou Ephessiou, 11527 Athens, Greece; p.char0ula@gmail.com (C.P.); etsirimon@bioacademy.gr (E.T.); jimsam5@hotmail.com (D.S.); kikigeo98@gmail.com (K.G.)

**Keywords:** neurofibromin, neurofibromatosis, astral microtubules, astrocyte, glioblastoma, spindle assembly, chromosome positioning

## Abstract

Mitotic spindles are highly organized, microtubule (MT)-based, transient structures that serve the fundamental function of unerring chromosome segregation during cell division and thus of genomic stability during tissue morphogenesis and homeostasis. Hence, a multitude of MT-associated proteins (MAPs) regulates the dynamic assembly of MTs in preparation for mitosis. Some tumor suppressors, normally functioning to prevent tumor development, have now emerged as significant MAPs. Among those, neurofibromin, the product of the Neurofibromatosis-1 gene (*NF1*), a major Ras GTPase activating protein (RasGAP) in neural cells, controls also the critical function of chromosome congression in astrocytic cellular contexts. Cell type- and development-regulated splicings may lead to the inclusion or exclusion of *NF1*exon51, which bears a nuclear localization sequence (NLS) for nuclear import at G2; yet the functions of the produced NLS and ΔNLS neurofibromin isoforms have not been previously addressed. By using a lentiviral shRNA system, we have generated glioblastoma SF268 cell lines with conditional knockdown of NLS or ΔNLS transcripts. In dissecting the roles of NLS or ΔNLS neurofibromins, we found that NLS-neurofibromin knockdown led to increased density of cytosolic MTs but loss of MT intersections, anastral spindles featuring large hollows and abnormal chromosome positioning, and finally abnormal chromosome segregation and increased micronuclei frequency. Therefore, we propose that NLS neurofibromin isoforms exert prominent mitotic functions.

## 1. Introduction

The ability of tubulins to rapidly form highly dynamic noncovalent polymers, the microtubules (MTs), has bestowed on them essential functions for the constant yet ever changing needs for spatial organization of cells, in order to execute critical processes, such as attaining function-coupled shapes, directed intracellular transport, cell migration, and the most important for development and maintenance of an organism cell divisions with accurate genomic transmission. For the latter, several types of MTs organize, elongate, and orient a bipolar spindle, through which chromosomes will position at the spindle equator for faithful sister chromatid separation and then segregation to the two daughter cells [1,2,3]. At least three major types serve these purposes, namely, astral MTs radiating from the centrosomes to position and orient the spindle through a protein machinery anchored to the cell cortex, microtubule bundles to link chromosome kinetochores to spindle poles (K-fibers), and interpolar bundles to elongate the spindle [4,5,6].

Accordingly, multitudes of structurally different proteins associate with MTs (MT-associated proteins, MAPs) to regulate their nucleation, polymerization, organization, bundling, and crosslinking in preparation for and completion of mitosis [7]. The availability of mitotic MAPs is tightly regulated by coordinated transcription, as well as by cell cycle-dependent post-translational modifications, most often phosphorylations that control protein trafficking, homeostasis, and inter- or intramolecular interactions [8,9,10,11]. As aberrations in any of these processes may lead to aneuploidy and further on to tumorigenesis, the ability of MAPs to modulate MT dynamics is recognized for its prognostic value in cancers, and as a sensible target for manipulations of microtubule-targeting cancer chemotherapy agents [12,13].

In this context, several MAPs have been described as oncogenes or as tumor suppressors and correspondingly several proteins, identified as such, were found to function as MAPs. Evidently, knockdown of tumor suppressor genes, such as adenomatous polyposis coli (*APC*) [14], *PTEN* [8], *NF2*, [15], *BRCA1* [16], *RASSF1A* [17], *NF1* [18], and *TP53* [19,20], often leads to defective mitotic spindle assembly and chromosome segregation errors. Given the usually compromised ability for DNA repair and the increased replication stress in these genetic backgrounds, the resulting aneuploidy may additionally feed chromosomal instability (CIN) [14,16,21,22] and thus rapid evolution of karyotypes with clonal expansion advantages and tumorigenesis [23,24,25,26,27,28]. Another typical characteristic of such tumor suppressors is the presence of a nuclear localization signal (NLS) in their amino acid sequence, which regulates both their timely nuclear import in preparation of mitosis and their release during spindle assembly [29,30,31].

**Scheme 1:**
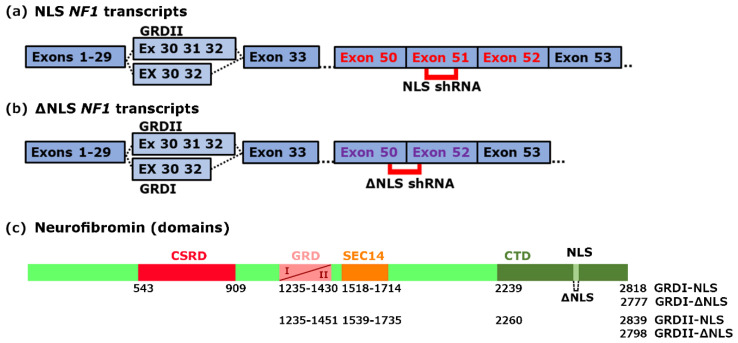
Schematic representation of *NF1* transcripts and neurofibromin domains. (**a**) and (**b**) depict alternative spliced events in the *NF1* gene that produce *NLS* and *ΔNLS* transcripts and indicate the sequences that are targeted by the used shRNAs. (**c**) Neurofibromin domains are depicted in proportionate size and location: **CSRD,** Cysteine/Serine-Rich domain; **GRD** GAP-related domain (RasGAP); **SEC14** Yeast Sec14p-like domain; **CTD** C-Terminal domain and **NLS** Nuclear localization sequence. Numbering of amino acids throughout the text corresponds to GRDI-NLS neurofibromin (Ensemble transcript NF-201).

We have recently shown that neurofibromin functionally belongs in the group of tumor suppressors with MAP properties that localize on the spindle and regulate chromosome congression at the metaphase plate [18]. More specifically, association of neurofibromin with MTs was first established by confocal microscopy and the molecule was proposed to act as a MAP through a small segment (residues 815–834), bearing in silico homology to MAP2 and Tau [32]. Since then, additional approaches, including co-immunoprecipitations, co-purifications, in vitro microtubule assembly, and affinity precipitations, have further documented this interaction [18,33,34,35,36]. Unfortunately, the full length cDNA, encoding this large, multidomain, and multifunctional protein (Scheme 1), has not been possible to obtain in a plasmid, and structural information remains limited to the RasGAP-related domain (GRD) [37] and the Sec-PH domain [38].

Neurofibromin is encoded by the *NF1* gene, mutations of which cause Neurofibromatosis type 1 (NF-1), a common, complex multisystem, familial cancer predisposition syndrome [39]. Neurofibromin is ubiquitously expressed early in development, while in the adult remains prominent in neural cells, namely, neurons, astrocytes, and Schwann cells; hence the most severe symptoms of NF-1 stem from these cell types ([39,40] and refs therein). In particular, high grade gliomas (anaplastic, glioblastoma-GBM) are more frequent in adults with NF-1, which have five times greater risk for GBM than the general population [39]. Moreover, *NF1* is the fifth most commonly mutated gene in sporadic glioblastoma [41]. This great mutation rate of the *NF1* gene that has made its cloning impossible, is highlighted by the 2800, most often truncating or nonsynonymous, different mutations identified to date, yet with few genotype–phenotype correlations postulated [42,43]. Confirmation of causative mutation with molecular diagnosis, a task complicated by 15 pseudogenes and no mutational hot spots, does not offer help for prognosis or treatment and the challenge to correlate genotype-phenotype in this disease of uncontrolled cell growth and tumorigenesis remains largely unmet.

The quest for genotype-phenotype correlation is complicated by developmental stage- and cell type-specific alternative splicing events of the *NF1* gene. Exon 31 (former 23a) is skipped in CNS neurons early on (transcript GRDI), whereas it is retained in astrocytes (GRDII) [44]. This exon corresponds to the center of the RasGAP-related domain (GRD) of neurofibromin, through which neurofibromin inactivates Ras. Due to the central role of Ras in many cellular functions and in carcinogenesis, GRDs have received high attention; they are functional RasGAPs when overexpressed in vitro [45] and, as we showed, in vivo [34], but no significant rescue capacity of GRDs alone has been shown for NF-1 tumor paradigms [46,47]. Instead, and along this direction, the importance of other neurofibromin domains (Scheme 1) in the allosteric regulation of GRD, has been established. Indeed, collective experimental evidence after overexpression of specific domain combinations has postulated that neurofibromin domains may bind each other [48], as well as, multiple proteins to coordinate Ras signalling [34,46,47,49,50]. For example, in glioblastoma cells stably overexpressing the N-terminus half (Cysteine/Serine Rich domain (CSRD) plus GRD), PKC-dependent phosphorylation of CSRD increases interactions with the actin cytoskeleton to regulate the Ras-GAP activity of GRD and suppresses Ras-dependent proliferation [49]. The clinical significance of these findings was directly postulated when large cohorts of NF-1 patients, heterozygous for nonsynonymous mutations of a five-amino acid stretch in the CSRD, were found to have high, >50%, predisposition to malignancies as compared to the general NF-1-affected population [42,43].

Exon 51 (former 43) in the C-terminus domain of neurofibromin (CTD) may also be alternatively transcribed; in the corresponding sequence of 41 amino acids (2518–2559) lies centrally a bipartite NLS sequence (2534–2550). We first identified this NLS in silico and documented experimentally that most neurofibromin molecules reside in the nucleus of neural cells [51]. Later genetic analysis revealed that NLS-*NF1* transcripts are highly expressed in the tissues in which neurofibromin expression remains high in the adult and which are implicated in NF-1- pathology, that is in neural tissues [52]. In subsequent studies on the function of the NLS in primary astrocytes and glioblastoma cells [18], which primarily express NLS transcripts, we provided the mechanism and purpose for a regulated nuclear import of endogenous, full length neurofibromin during the cell cycle: a. at late S phase neurofibromin synthesis increases, and PKCε-phosphorylations on the NLS-adjacent Ser2808 mobilize the molecule to shuttle by the Ran/importin system that requires an NLS into the nucleus (now also confirmed in cancer breast cells [53]); b. neurofibromin localizes on centrosomes and on the spindle throughout mitosis; and c. siRNA-depletion of all *NF1* transcripts leads to errors in chromosome congression. As further established by phospho-ablating or -mimetic constructs of CTD + NLS [18], only NLS isoforms have the ability to shuttle in and out the nucleus and therefore these congression errors may be attributed mostly to depletion of NLS-neurofibromin. However, beyond these studies, no other information exists on the properties and functions of NLS or ΔNLS neurofibromin isoforms.

Therefore, we have launched a program to genetically manipulate cells to express either NLS or ΔNLS transcripts, and thus neurofibromin isoforms, using transcript-specific shRNAs, and have now addressed whether inclusion or not of the amino acids encoded by exon 51, suffices to produce a different MAP.

## 2. Materials and Methods

### 2.1. NF1 Transcript Knockdown with shRNA Lentiviruses

In order to observe the effects of NLS- and ΔNLS- neurofibromin isoforms after knockdown of their respective *NF1* transcript (Scheme 1) over a long time and many cell divisions while increasing reproducibility of results, we chose to interfere with the mRNA production of NLS and ΔNLS *NF1* transcripts [52], by generating for each transcript shRNA constructs, which are capable of DNA integration [54]. Thus, short hairpin RNAs against sequences within exon 51 or against the exon 50–52 junction sequences (Ensemble, ENSG00000196712) were designed in order to degrade NLS or ΔNLS *NF1* transcripts (Scheme 1a,b, respectively, using the Biosettia (San Diego, California, USA), Invivogen (Toulouse, France) and Hannon Lab (CRUK Cambridge Institute, UK) (GSHL) platforms and synthesized by Macrogen (Seoul, Korea). Each target sequence was used in a complementary pair of oligos where the sense and antisense sequence of interest formed the 21 nt stem of the shRNA. The oligos also bore flanking miR30 sequences and a 6 nt loop [55].

Specifically, the shRNA oligos were used to generate the insert shRNA fragment by the annealing method, which allowed us to subclone it into the XhoI/EcoRI- digested lentiviral, tet-inducible RFP-vector pINDUCER10 backbone [56] (Addgene, LGC Standards, Teddington, UK) and produce the RFP-Tet-NLS-pINDUCER10 and RFP-Tet-ΔNLS-pINDUCER constructs. For lentivirus production, HEK293T cells, grown to 60% confluency in Dulbecco’s modified Eagle medium (DMEM, ThermoFisher, Waltham, MA, USA) and 10% fetal bovine serum (FBS, Biowest, Nuaille, France) were co-transfected with the generated plasmids or mock plasmid, along with the VSV-G (pMD2.G, Addgene) and gag-pol (pCMV-dR8.91, Applied Biological Materials Inc., Richmond, BC, Canada) expression plasmids. Lentivirus-containing medium was collected after 48 h, spun at 4500 rpm for 20 min, filtered through a 20 μm syringe filter and used to transduce SF268 cells (passage 60, ATCC, LGC Standards GmbH, Germany) for 48 h, and then the medium was replenished with fresh medium for another 24 h. At this point cells were treated with 10 mM of doxycycline to induce shRNA expression and with 5 μM puromycin to select transduced cells and generate cell lines stably expressing the candidate shRNAs. A week later cells were sorted (mean ± 1 standard deviation) using Fluorescence Assisted Cell Sorting (FACS Facility, BRFAA), and banked at different passages in 5% DMSO, 10% glycerol, 20% DMEM, and 65% FBS at −135 °C. All cell lines were maintained in culture with 10% FBS, 2 mM l-glutamine and 1% penicillin/streptomycin at 37 °C and 5% CO_2_.

**Figure 1:**
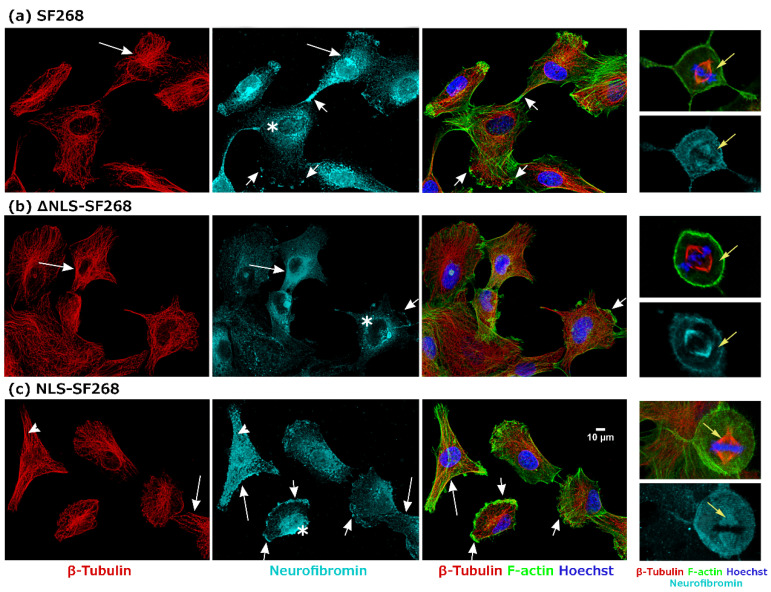
Subcellular localization and co-localization with β-tubulin or F-actin of endogenous neurofibromins differ in cells genetically modified with shRNA knockdown of non-NLS or NLS *NF1* transcripts and express either ΔNLS or NLS neurofibromin, respectively. Asynchronous cells, treated with the cross-linker DSP, extracted with Triton X-100, and fixed with PFA, were quadruply stained for β-tubulin (red), neurofibromin (cyan), F-actin (green), and chromatin (blue), as described in Methods. Panels show representative confocal deconvolved images at interphase (first three columns) and at metaphase (last column) of (**a**) SF268 naïve cells, (**b**) ΔNLS-SF268 cells, and (**c**) NLS-SF268 cells. Detailed description is provided in the text. Images are the maximal intensity projection of 0.34 μm confocal planes that include all F-actin and β-tubulin signals; representative single planes and corresponding fluorochrome correlation scattered plots are presented in Supplementary Figure S2.

Transcript and protein knockdown was confirmed with immunofluorescence for ∆NLS isoform nuclear expression (e.g., Figure 1), and with qPCR and Western blotting (WB) for both ∆NLS and NLS transcript and isoform expression (e.g., Supplementary Figure S1a,b). More specifically, total RNA from 4 × 10^6^ cells from each cell line post FACS sorting was isolated with TRI Reagent (Sigma, St. Louis, MO, USA), and reverse transcribed with M-MuLV Reverse Transcriptase (Genscript, Piscataway, NJ, USA) and random primers (Invitrogen, Carlsbad, CA, USA), as previously described, e.g., [18,57]. Every sample within an experiment was reverse transcribed at the same time and cDNAs were stored at –80 °C until used. One hundred ng of cDNA were used in a 10 μL reaction mixture, consisting of NLS (5′-GAAGTTGCTTGGAACAAGGAAAAGT-3′, 5′-TCATAATCAGTTTCTGCTACTCTCC-3′) or ΔNLS (5′-GAAGTTGCTTGAAACTCAGAGGA-3′, 5′-AACACAACACTGGCCTCTGCTAA-3′) specific primers at 0.5 μΜ and Kapa SYBR Fast Universal qPCR Master Mix (Roche, Switzerland). Empty vector DNA was used as a non-template control. Quantitative PCR (qPCR) was performed using the Roche LightCycler^®^ 96 System (Basel, Switzerland), under the following conditions: preincubation at 95 °C × 5 min, amplification for 45 cycles at 95 °C × 15 s and then at 60 °C × 60 s, and melting at 95 °C × 10 s, 65 °C × 60 s, and 97 °C × 1 s. *GAPDH* was used as reference gene. Reactions were set up from all three cell lines in triplicate from at least three separate experiments corresponding to different cDNA isolation dates and data were analyzed using the relative quantification 2-ΔΔCT method [58].

Western blot analysis was essentially done as we have previously described [18,49,57,59,60]: equal amounts (60 μg) of total cell homogenates in radioimmunoprecipitation (RIPA) buffer, supplemented with protease and phosphatase inhibitors, were resuspended in 5× Laemmli buffer and proteins were separated with Sodium dodecyl sulfate-polyacrylamide gel electrophoresis (SDS-PAGE). After electrophoresis, proteins were transferred into nitrocellulose membranes and probed with primary antibodies. Immunoreactivity was visualized with the appropriate species-specific antibody conjugated to horseradish peroxidase (HRP), enhanced chemiluminescence, and film exposure; films were then scanned and densitometry analysis was performed with the ImageJ software.

Using this methodology, we chose the following sequences as both fulfilling optimal criteria and efficiently reducing by 85–95% mRNA levels: for the NLS *NF1* transcript ^7609^GAAATGGAATCAGGGATCACA^7629^ and for the ∆NLS ^7546^TGCTTGAAACTCA GAGGATTTCC^7552^, respectively (Supplementary Figure S1a); the 9:1 ratio [18] of GRDII:GRDI was not affected (not shown). As expected, total neurofibromin protein levels, assessed by densitometric analysis (ImageJ) of WBs using polyclonal antibodies sc-13023 and H-300 (Santa Cruz Biotechnology, Heidelberg, Germany), decreased by 30% in ΔNLS-SF268 and by 18% for NLS-SF268. Almost identical results for each isoform were obtained after silver staining of immunoprecipitated neurofibromin, albeit using different antibodies, all raised against the N-terminus of neurofibromin: rabbit polyclonals sc-68 and sc-13032 recognized neurofibromin in lysates of ∆NLS cells, but only partially in NLS cell lysates, whereas the monoclonal sc-20016 recognized NLS isoforms at levels compatible with WB results, but ∆NLS isoforms only faintly (Supplementary Figure S1c).

### 2.2. Immunocytochemistry

Immunofluorescence analyses were performed as previously described by us [18,51,60]. Briefly, cells were plated on glass coverslips and grown to desired confluency; prior to a 10 min extraction with 0.2% Triton X-100 and fixation with paraformaldehyde (PFA), cells were treated with the protein crosslinker 3,3′-dithiodipropionic acid di(*N*-hydroxysuccinimide ester) (DSP, ProteoChem, Hurricane, UT, USA) in order to best visualize cytoskeleton and associated proteins. In some experiments, extraction with 3 min exposure to 0.1% Triton X-100 was performed after PFA fixation and in others, coverslips were immersed in −20 °C methanol for 10 min, all prior to blocking with 3% appropriate animal sera and exposure to primary antibodies for 3 h. Primary antibodies used for detection of: neurofibromin, namely mouse monoclonals sc-376886 and sc-20016, and the polyclonals sc-68 and sc-13032 were purchased from Santa Cruz; β-tubulin, mouse monoclonal T4026 was from Sigma (St Louis, MO, USA); and γ-tubulin, ab11316 mouse monoclonal from Abcam (Cambridge, UK). Visualization of primary antibody binding was accomplished using the appropriate secondary antibodies conjugated to Texas Red^®^ (GeneScript, Piscataway, NJ, USA), Cy™5, or fluorescein (Jackson ImmunoResearch, Pennsylvania, USA); F-actin was visualized with Oregon Green™ 488 phalloidin (ThermoFisher, Waltham, MA, USA) and chromatin structures with Hoechst 33258 (Sigma). When indicated, cells were synchronized with a double thymidine block, as we have previously described [18].

### 2.3. Imaging and Image Analysis

#### 2.3.1. Fluorescence

Fluorescence was imaged using a Leica TCS SP5 inverted confocal microscope with a motorized stage, a 63×/1.4 NA HC PL APO CS oil lens, and a Tandem Scanner (Leica Microsystems, Mannheim, Germany; Biological Imaging Unit, BRFAA), and z-stacks with 0.34 μm-thick z optical sections were captured for each fluorophore. When appropriate, images were connected to an extended focus single section, using the maximal intensity projection algorithm of the LAS AF Software (Leica Microsystems). For every experiment, samples from all three cell lines were imaged using identical microscope settings, which were set prior to capturing and after pilot screening to define the common settings at which no saturated pixels were detected in any of the samples. All images were deconvolved using the Richardson-Lucy total variation algorithm found in DeconvolutionLab2 plugin [61]. Theoretical Point-Spread Functions (PSFs) were created using the Born & Wolf PSF model [62] from the PSFGenerator plugin.

#### 2.3.2. Phase Microphotographs

Phase microphotographs of the wound healing assays were captured using a Hamamatsu Orca-ER CCD camera, connected to a Zeiss 200M inverted microscope equipped with a motorized stage, all controlled with the Slidebook^TM^ 6.0 software (3i, Göttingen, Germany) and analyzed using the ImageJ public domain software (NIH).

#### 2.3.3. Colocalization Analysis

Colocalization analysis of β-tubulin, F-actin or γ-tubulin with neurofibromin was performed with the Volocity^®^ 6.1.2 software (Perkin-Elmer, Seer Green, UK), using the Costes Pearson’s Correlation Coefficient (PCC) algorithm [63]. Regions of interest (ROI) were drawn manually around a. cells for β-tubulin/neurofibromin b. cells or lamellipodia for F-actin/neurofibromin, and c. centrosomes for γ-tubulin/neurofibromin colocalizations. Within ROIs, this software applies thresholding [64], and then calculates and scores the extent of overlap between fluorophores within a range of 1, if a perfect positive correlation, to -1, if a perfect but inverse correlation; 0 represents a random distribution. PCC means were automatically calculated from the generated scattered plots and exported for every single plane in an excel file; such examples are shown in Supplementary Figure S2 and correspond to the images shown in Figure 1.

#### 2.3.4. Fluorescence Intensity

Fluorescence intensity of β-tubulin signals from the spindle, or from astral or cytosolic MTs, and of γ-tubulin signals from centrosomes was measured using IMARIS^®^ 8.3.1 software (Bitplane, Zürich, Switzerland) in 3D image reconstructions. The IMARIS surface tool (0.2 μm Gaussian Filter) was used to render solid surfaces of spindles and centrosomes, regardless of cell orientation (e.g., Figure 4d) and measurements were based on the mean intensity, whereas volume was automatically calculated in μm^3^.

#### 2.3.5. Microtubule Intersection Analysis

**Figure 2:**
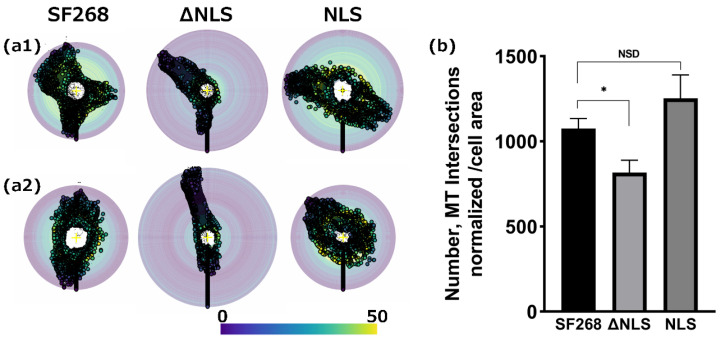
Knockdown of NLS neurofibromin reduces intracellular microtubule (MT) intersections. Sholl analysis on confocal images, captured after different fixations, (**a1**) DSP crosslinking and PFA fixation or (**a2**) methanol fixation, revealed that SF268 and NLS-SF268 cells showed high numbers (yellow hues) of MT intersections around the nucleus and the MTOC/centrosome area which gradually declined towards the cell periphery (yellow to light blue to purple hues), whereas ΔNLS-SF268 cells displayed lower numbers of MT intersections and almost uniform distribution along the nucleus-cortex radius. (**b**) Quantification of MT intersections in each genetic background, normalized per cell area. Bracketed bars represent the means and standard errors of measurements in 20 cells per cell line from each of 3 separate experiments; *, *p* < 0.05; NSD, no significant difference.

Microtubule intersection analysis was performed using ImageJ Sholl Analysis [65], an algorithm that creates a series of concentric circles around a specified focus (in this case the center of a nucleus, e.g., Figure 2) and then counts how many times microtubules intersect the sampling circles, on cells fixed with DSP and PFA or with methanol and immunostained for β-tubulin. The area of the nucleus, as defined from Hoechst fluorescence, was excluded from the analysis. Results are exported as both TIFF images, where each concentric circle is color-coded according to their Sholl profile and the detected number of intersections (yellow hues indicates highest number of intersections), and as numerical data in an excel file, for statistical analysis.

#### 2.3.6. Spindle Length and Orientation

Spindle length and orientation analysis was also performed using ImageJ in z-stacks of confocal planes of cells in metaphase. Spindle length was determined as the distance between the two centrosomes, visible after γ-tubulin immunostaining. For this, the two planes with the strongest signal from each pole were recorded, the number of intermediate planes between these two was added and the sum multiplied by 0.34 (plane thickness in μm) to calculate the side of an orthogonal triangle; the other side was the distance in μm, calculated as the distance of the poles on maximal intensity projection of these stacks, and the spindle length was its hypotenuse. The angle was then calculated by the cosine between the side of z-stack thickness and the hypotenuse.

#### 2.3.7. Astral Microtubule Length

Astral microtubule length analysis was performed using the ImageJ plugin Single Neurite Tracer [66]: each visible astral microtubule was traced across z-stack in a semi-automated manner, that is, after manually defining the starting and ending point of microtubules. When preparing maximal intensity projections for Figures depicting spindles, e.g., Figure 4, astral microtubules were not readily visible without enhancing the fluorescence to points that the rest of the spindle was oversaturated with color. Therefore, the astral microtubule area is presented with enhanced brightness in Supplementary Figure S4.

### 2.4. Wound Healing Assay

For the wound healing assay, cells at 60–70% confluency were detached by trypsin treatment and seeded on glass bottom culture dishes (MatTek, Ashland, MA, USA). When cells formed a monolayer (≥90% confluency), a wound was made by scratching a line with a white tip across the bottom of the dish. Cells were rinsed gently with PBS, medium was replenished and cultures were photographed in several areas under phase in a Zeiss 200M inverted microscope; coordinates of these areas were marked with the appropriate stage Slidebook^TM^ tool and the same areas were photographed again after 24 h. Slidebook^TM^ images, exported as TIFFs, were then used to calculate the wound area by manually tracing the cell-free area in captured images using ImageJ. For analysis of the microtubule-organizing center (MTOC) and nucleus positions during migration, cells cultured on coverslips were immunostained with β-tubulin and Hoechst, respectively, as described above. For time-lapse video microscopy, cells were resuspended in 35 mm glass bottom dishes (MatTek) and left to grow into a monolayer, in normal CO_2_ and temperature. The monolayer on the glass part of the dish was scratched, as described above. After 14 h, the cells were rinsed and their medium was replaced by Leibovitz’s L15 medium without phenol red (ThermoFisher); phase photographs were shot every 2 min for an observation period of 4 h and the results were compacted as a video with the appropriate Slidebook^TM^ tool.

### 2.5. Statistics

All experiments were performed five to ten times with similar results and numerical data were analyzed by ANOVA, using the GraphPad Prism 8 software and a set statistical significance level (*p*) of 0.05. Graphs were generated with the same software and images were organized using Adobe Photoshop.

### 2.6. Databases

The following databases have been used: genome browser; https://www.ensembl.org/protein alignment; https://www.ebi.ac.uk/Tools/psa/emboss_water/ (Cambridge, UK); and prediction of serine, threonine or tyrosine phosphorylation sites, NetPhos 3.1 Server http://www.cbs.dtu.dk/services/NetPhos (DTU Bioinformatics, Lyngby, Denmark).

## 3. Results

### 3.1. Differential Subcellular Distribution of Neurofibromin NLS and ΔNLS Isoforms and Associations with the Actin and Tubulin Cytoskeletons affect Cytosolic MT Organization and Cell Migration Patterns

To further address the mechanism by which neurofibromin regulates chromosome congression [18] and considering together that a. the major cellular target in NF-1 for abnormal proliferation and carcinogenesis is the astrocyte ([39,40], b. neurofibromin accumulates in the nucleus in a Ran-dependent manner at late S/G2, and c. the higher expression of NLS- over ΔNLS *-NF1* transcripts in astrocytes, we sought to evaluate separately the roles of ΔNLS- and NLS-isoforms in spindle assembly in an astrocytic cell context. Thus, we generated SF268 glioblastoma cell lines that stably express, under the control of doxycycline, shRNAs specifically designed to degrade either the GRDI- or GRDII-ΔNLS transcripts or the GRDI- and GRDII-NLS-*NF1* transcripts (Scheme 1; for simplicity, we will refer to those as NLS-SF268 cells and ΔNLS-SF268 cells, respectively). Because we have previously established colocalization of endogenous neurofibromin with the microtubule (MT) and F-actin cytoskeletons [49,51,60], we first proceeded with confocal image analysis of cells immunostained for β-tubulin and neurofibromin and co-stained for filamentous actin with FITC-phalloidin and chromatin/chromosomes with Hoechst 33258. Our analyses included intensity assessment of β-tubulin and neurofibromin fluorescence signals using IMARIS, and β-tubulin/neurofibromin and F-actin/neurofibromin colocalization quantitation using Volocity, as described in Materials and Methods. In order to best preserve filamentous cytoskeleton structures and their associated proteins, cells were treated with the crosslinker DSP and then extracted with Triton X-100 prior to fixation [18,51,67].

Parental SF268 cells, which express both NLS- or ΔNLS-isoforms, typically [18] show pools of endogenous neurofibromin to colocalize with cytoplasmic microtubules and the distinctly organized MTOC (Figure 1a, long arrows), and the mitotic spindle (Figure 1a, yellow arrows), as well as with F-actin, mainly at the cell cortex and lamellipodia (Figure 1a, small arrows). About 35% of neurofibromin immunofluorescence derives from the nucleus (Figure 1a, asterisk), part of which is contributed by the nuclear envelope through interactions with the nuclear intermediate filament lamin A and the rest from associations with non-chromatin nuclear structures [18]. As expected, nuclear localization greatly differed among the genetically modified cell lines, with ΔNLS neurofibromins practically absent from the nucleus of ΔNLS-SF268 cells at interphase, while NLS isoforms (NLS-SF268 cells) retained various intensity levels in the nucleus, as seen in the parental cells (asterisks in Figure 1b versus a and c; Table 1).

Moreover, in cells depleted of NLS neurofibromins and thus expressing only ΔNLS neurofibromins (ΔNLS-SF268), association of neurofibromin with F-actin was significantly limited, especially in lamellipodia that appeared less developed, as well (Figure 1b, small arrow and Table 1). In contrast, in their counterpart NLS-SF268 cells, the NLS-neurofibromin richly decorated their well-formed lamellipodia and significant increases of association with F-actin were recorded (Figure 1c, small arrows and Table 1). Association with tubulin was not significantly reduced in ΔNLS-SF268 cells, yet neurofibromin localization appeared more diffused without the typical fibrillar patterns and perinuclear concentrations (Figure 1b, long arrows and Table 1). Notably, a cytoplasmic MTOC was not readily recognized and MTs organized a dense yet non-radial network of often parallel fibers, with significantly increased β-tubulin intensity (Table 1). To the contrary, NLS neurofibromin colocalization with β-tubulin was significantly enhanced (NLS-SF268; Figure 1c, arrowheads; Table 1), while β-tubulin intensity was not affected (Table 1). Both types of neurofibromins retained colocalization with β-tubulin on the mitotic spindle (Figure 1, yellow arrows in far-right column panels and Supplementary Figure S2d), albeit colocalization levels with NLS-neurofibromin were significantly raised, as also seen with MTs in the cytosol (Table 1). Taken together, these data showed that NLS-neurofibromins exhibit greater affinity for spindle MTs and for cytosolic MTs without affecting their density, whereas ΔNLS-neurofibromins, exhibiting lesser affinity for cytosolic MTs, inversely regulated MT densities in the cytoplasm.

To substantiate the differential patterns of MT organization and β-tubulin densities, we applied Sholl analysis (ImageJ) in order to quantitate numbers and dispersion of MT intersections within each *NF1*-type cell. Both parameters differed among the ΔNLS-SF268 cells and the parental or NLS-SF268 cells (Figure 2). Specifically, numbers of MT intersections in SF268 and in cells expressing NLS neurofibromin were higher in the perinuclear area (inner yellow circles, Figure 2a left and right panels) and gradually reduced towards the cell periphery (green to purple circles), independent of the fixation used (Figure 2 row a1, DSP and Triton X-100 extraction; row a2, methanol). In contrast, numbers of intersections in ΔNLS-SF268 were overall significantly less, with the gradient of high-to-low concentrations (yellow to purple hues) from the perinuclear area towards the cortex significantly toned down (Figure 2a middle panels and b), when compared to those seen in parental SF268 and NLS-SF268 cells. Again, similar results were obtained with DSP or methanol fixation (Figure 2, middle panel in a1 versus a2 row), further corroborating the images of less radial or tufted arrangements of intracellular MTs in ΔNLS-SF268 cells (Figure 1b).

**Figure 3:**
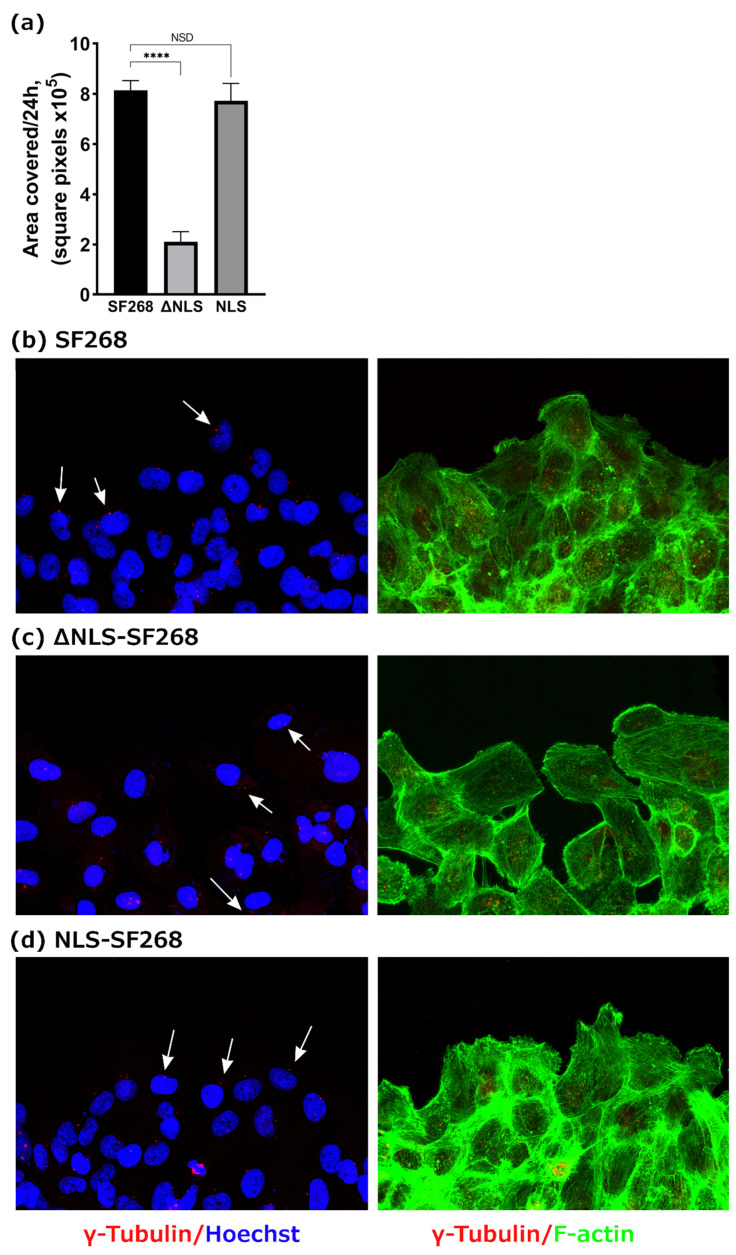
Knockdown of NLS neurofibromin imposes changes in orientation of MTOCs and migration patterns of glioblastoma cells. (**a**) Quantification of the wound healing assay at 24 h. The wound area was calculated by manually tracing in captured images at 0 and 24 h post-scratching and data were analyzed by ANOVA and Tukey post hoc test. Bracketed bars represent the mean and standard error of five different experiments per cell line; ****, *p* < 0.0001, NSD, no significant difference. Comparisons of centrosome position (arrows) in fixed (DSP + PFA), immunostained with γ-tubulin, (**b**) parental SF268, (**c**) ΔNLS-SF268, and (**d**) NLS-SF268 cells revealed misorientiation of both centrosomes and leading edges, relative to the nucleus (chromatin stained with Hoechst) and the long axis of the wound (upper part of panels), with loss of NLS neurofibromins. Images are the maximal intensity projection of confocal serial 0.34 μm-thick Z-sections with γ-tubulin and Hoechst signals (~10 µm).

We then investigated the different robustness of MTOC formation among ΔNLS-SF268 cells and the parental or NLS-SF268. In epithelial cells, in particular astrocytes, a most well explained paradigm of cellular polarization [68] is the relocation of the centrosome, the major MTOC, between the nucleus and the leading edge of the cell during migration, most likely in order to help provide needed, Golgi-processed proteins to the cell front along microtubules. Thus, to assess whether the ability of cells to distinctly organize and polarize their MTOCs during cell migration was affected by the expression of NLS or ΔNLS neurofibromins, we used wound healing assays. Parental and genetically modified cells, grown on glass-bottom MatTeK 35 mm dishes as monolayers, were scratched with a pipette tip to create a linear wound and photographed at this time and again 24 h later under phase-contrast (Supplementary Figure S3). This analysis showed that NLS-SF268 retained similar migration progress to parental, while ΔNLS-SF268 cells would cover only 25% of that covered by the parental cells or NLS-SF268 (Figure 3a). When positions of nuclei and centrosomes were observed in fixed cells, stained for γ-tubulin and Hoechst 12 h post-scratching, confocal microscopy revealed that in parental and NLS-SF268 cells centrosomes were readily positioned between the leading edge and the nucleus and directed towards the long axon of the wound, whereas ΔNLS-SF268 cells had randomly polarized centrosomes in relation to these points of reference (Figure 3, arrows in c versus b and d).

Moreover, directional movement studies (post-scratching time-lapse video microscopy, Supplementary Videos S1–S3) confirmed that NLS-SF268 cells are highly migratory and, like the parental SF268, move with a directed, multicellular movement, often following a leader cell [69]. In contrast, ΔNLS-SF268 cells moved almost as fast in terms of speed, but in a palindromic motion, thus failing to repair the “scratch wound” even after 48 h. This is the first time that inclusion or not of an NLS in the neurofibromin sequence is linked to modifications of astrocytic cell migration, and, at least for ΔNLS neurofibromin, to defective centrosome positioning and cell polarity.

### 3.2. Loss of NLS-Neurofibromin Leads to Defects in Spindle Length and Geometry, and Chromosome Congression at Metaphase

**Figure 4:**
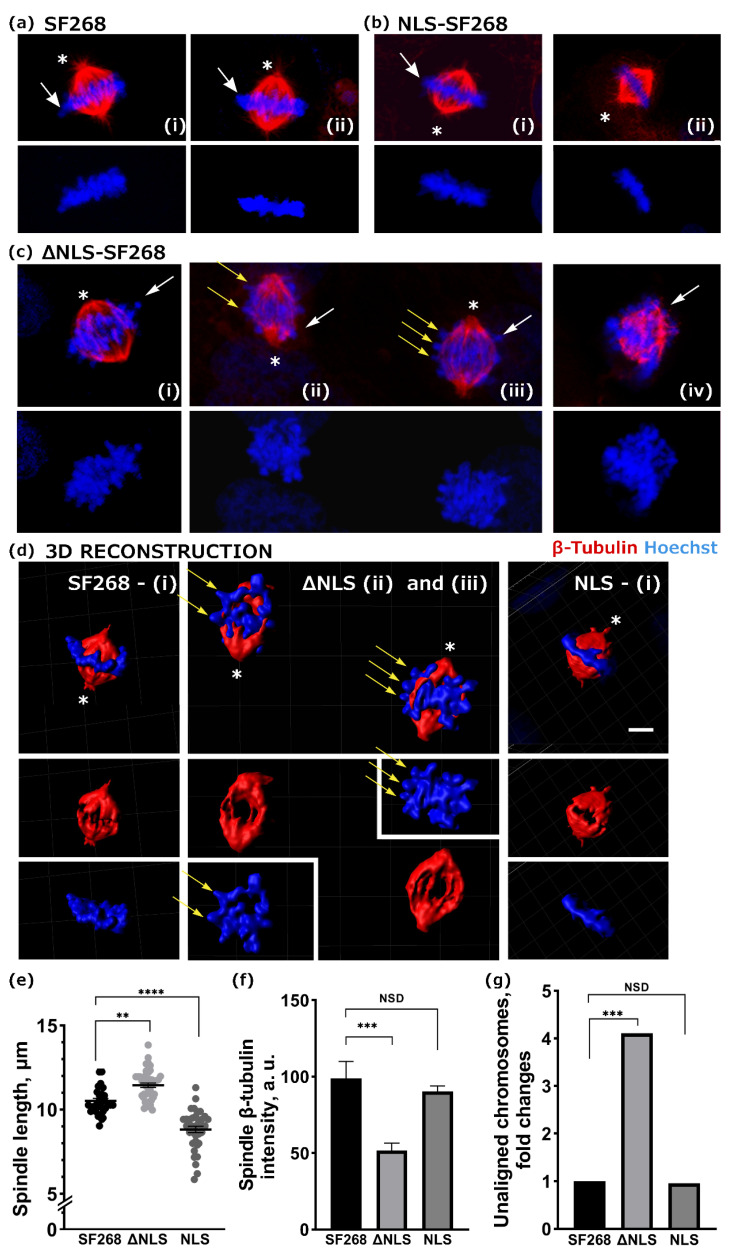
Spindle MT properties and configuration, and chromosome congression are regulated by NLS neurofibromin isoforms. Immunofluorescence confocal images of synchronized cells in metaphase treated with DSP and stained for β-tubulin (red) and chromatin (blue) of (**a**) SF268, (**b**) NLS, and (**c**) ΔNLS-SF268 cells. SF268 parentals and NLS-SF268 cells have astral MTs emanating from the spindle poles (asterisks) and proper chromosome congression at the spindle equator (white arrows). ΔNLS cells lack astral MTs (asterisks) and display abnormal chromosome congression (yellow arrows); Latin numerals in panels label individual spindles. (**d**) 3D reconstructed confocal images of metaphasic cells, using IMARIS. Yellow arrows show abnormal chromosome congression and asterisks astral MTs (Latin numbers indicate identical spindles shown in a–c). Bar = 5 µm in all panels. (**e**) Quantification of spindle length in each genetic background, measured as maximal pole-to-pole distance and (**f**) of β-Tubulin intensities in spindles, measured using IMARIS; bracketed bars represent the means and standard errors of measurements of 30–40 spindles per cell line from each of 3 separate experiments. (**g**) Percentage of spindles in metaphase with aberrant chromosome congression, expressed as fold change over parentals, calculated from a 200 metaphases per cell line from each of 3 different experiments. *p* values for all graphs, based on $\bar{x}$ ± SE are: ** ≤ 0.01, *** ≤ 0.001, **** < 0.0001, and NSD, no significant difference in ANOVA (see also Supplementary Figure S4).

As described earlier, at late S phase, NLS-*NF1* transcript levels increase along with increases in neurofibromin abundance; the molecule then mobilizes, after PKCε-phosphorylation on the NLS-adjacent Ser2808, to bind to importins and accumulate in the nucleus, and to localize on the spindle throughout mitosis. Moreover, effective depletion of neurofibromin (>96% of protein), with an siRNA against exon 4 and thus all transcripts, causes aberrant chromosome congression with chromosomes remaining at various distances away from the metaphase plate [18]. Therefore, we next examined which type, NLS or ΔNLS-neurofibromin, was causally linked to this phenotype. For best results, we first established the average spindle length for each modified genotype and assessed congression phenotypes in relation to spindle length (Figure 4a–g). In pilot experiments, we established that the percentage of cells with a spindle axis almost perpendicular (vertical) to the surface of the coverslip was strictly inversely related to confluency for all, naïve or genetically modified, SF268 cells, therefore all described measurements were performed at 70–80% confluency, when >90% of spindles are almost horizontal [70].

As shown in Figure 4a, parental cells at metaphase and fully developed metaphasic spindles (pole to pole distance $\bar{x}$ = 10.8 ± 0.2 μm; Figure 4e) had normally congressed chromosomes at the spindle equator (white arrows), as also did NLS-neurofibromin expressing cells (Figure 4b, white arrows), which, however, had significantly shorter spindles (maximum length $\bar{x}$ = 8.91 ± 0.22 μm, Figure 4e). In stark contrast, these parameters were inversely regulated in cells expressing only ΔNLS-neurofibromin, which had very poorly aligned chromosomes at the equator (Figure 4c, white arrows), even when the metaphasic spindle was fully developed, at least in length ($\bar{x}$ = 11.5 ± 0.15 μm, Figure 4e). More specifically, in over 50% of cells, the majority of chromosomes appeared in rows that formed a wide belt (Figure 4c, spindles ii–iv) and in the rest formed a better defined metaphase disk of chromosomes, still, however, lacking the tight alignment (e.g., Figure 4c, spindle i) observed in parentals.

Intensity measurements in three-dimensional reconstructions of all confocal z-planes with β-tubulin and Hoechst fluorescence signals (Figure 4d; reconstructed spindles are those shown in a.i for SF268, in c.ii and iii for ΔNLS, and in b.i for NLS cells) revealed that β-tubulin intensity in the anastral spindles of ΔNLS cells (Figure 4d, asterisks in SF268 and NLS versus ΔNLS cells) was significantly decreased (Figure 4f). Moreover, these 3D reconstructions strongly indicated that the low β-tubulin intensities were derived from lesser populations of polar MTs, as the spindle geometry was dramatically different, featuring large hollows by the equator and chromosomes forming queues on some prominent thick MT formations (Figure 4, yellow arrows). For quantification of chromosome congression, we counted spindles with lengths of $\bar{x}\;$ ± SE, relatively horizontal positions, and well formed metaphase disks (e.g., Figure 4c, spindle i), and found that more than 54% of cells had unaligned chromosomes (e.g., Figure 4c, white arrow), a statistically significant difference of over 4-fold as compared to control and NLS-SF268 cells (Figure 4g). These congression abnormalities, greater than those observed with ablation of all *NF1* transcripts [18], occurred within days of exposure to doxycycline, and thus expression of the NLS-*NF1* targeting shRNAs and knockdown of NLS transcripts and neurofibromin isoforms. Instantly noticeable was also the lack of astral MTs in all ΔNLS-SF268 cells (asterisks in Figure 4a and b versus c). All effects were reversed 10 days after removal of doxycycline from the culture medium. These results establish for the first time that NLS- and ΔNLS-neurofibromins actively participate in the formation of mitotic asters and spindles, possibly by exerting opposing effects, at least as the effects on the spindle length may indicate. Nonetheless, these data suggested that NLS neurofibromins are required for proper spindle assembly and chromosome congression, while pointing out that astral MT formation could be the earliest event to be compromised.

### 3.3. Neurofibromin NLS and ΔNLS Isoforms Differentially Regulate Aster Growth, Astral Microtubule Length, and Spindle Positioning

**Figure 5:**
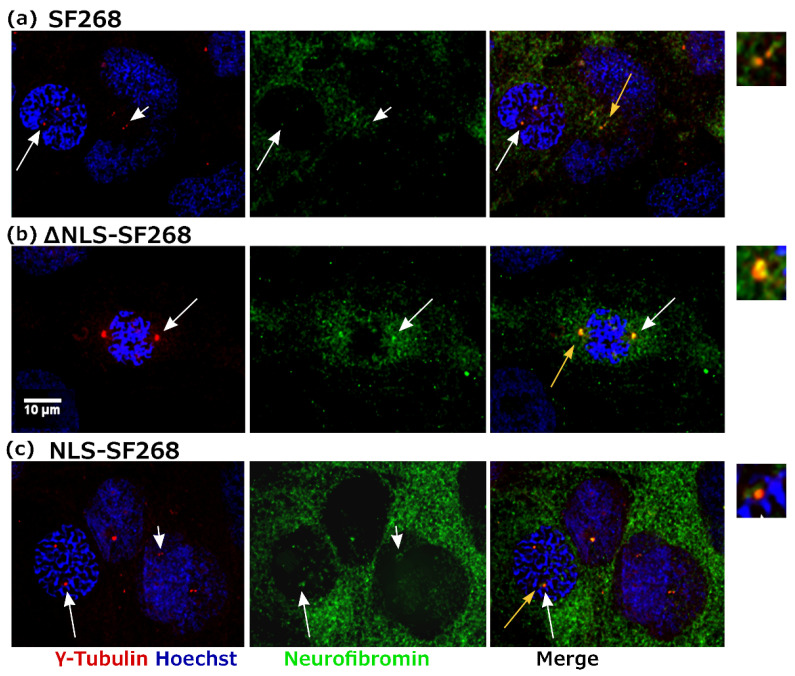
Differential neurofibromin isoform colocalization with γ-tubulin on mitotic centrosomes. (**a**) SF268, (**b**), ΔNLS and (**c**) NLS synchronized late S-G2 cells were fixed with PFA and immunostained with γ-tubulin (red) and neurofibromin (green); chromatin was stained with Hoechst (blue). Panels, representing maximal intensity projections of all stacks containing fluorescence signals, show colocalization of γ-tubulin and neurofibromin (long white and short arrows). Partial overlapping signals in parental SF268 and in NLS-SF268 cells, but not ΔNLS-SF268 cells are denoted with yellow arrows; the same centrosomes are shown at 300% magnification in the neighboring small panels.

We have previously demonstrated that endogenous neurofibromin localizes at the duplicated centrosome and co-immunoprecipitates with γ-tubulin, at least through its GRD and CTD domains, in astrocytes and glioblastoma cells [18,34]. Therefore, to address the role of neurofibromin isoforms in mitotic aster formation, we began our investigations with analysis of centrosomal features, during the transition from late S to M phases. Survey of cells at late S to late G2 and selection of cells with condensed chromosomes (early prophase) with IMARIS intensity analysis showed a statistically significant increase in the volume, as judged by γ-tubulin immunopositivity, of duplicated centrosomes in cells expressing ΔNLS neurofibromin, when compared to parental and NLS-SF268 cells (Table 2 and Figure 5, long white arrows in a and c versus b); abundance of γ-tubulin (not shown) and intensities (Table 2) were similar among all cell lines.

In terms of colocalization levels of NLS or ΔNLS neurofibromins with γ-tubulin on the duplicated centrosome, before (e.g., Figure 5, short arrows) or after centrosome separation (Figure 5, long white arrows), lesser amounts of NLS neurofibromin were recorded to associate with γ-tubulin as compared to ΔNLS neurofibromin (Table 2). Close inspection of the separated centrosomes in confocal images showed that, in NLS-SF268 cells and in parental cells, the neurofibromin-positive areas partially overlapped with those positive for γ-tubulin, whereas in ΔNLS cells, the γ-tubulin area was visibly larger (Figure 5, yellow arrows and small panels). While it is not possible at this level of analysis to interpret these patterns, in conjunction with the metaphase phenotypes (Figure 4), these data altogether indicated that potentially NLS neurofibromins contribute to the formation of a more efficient mitotic centrosome, in terms of future spindle assembly and function.

**Figure 6:**
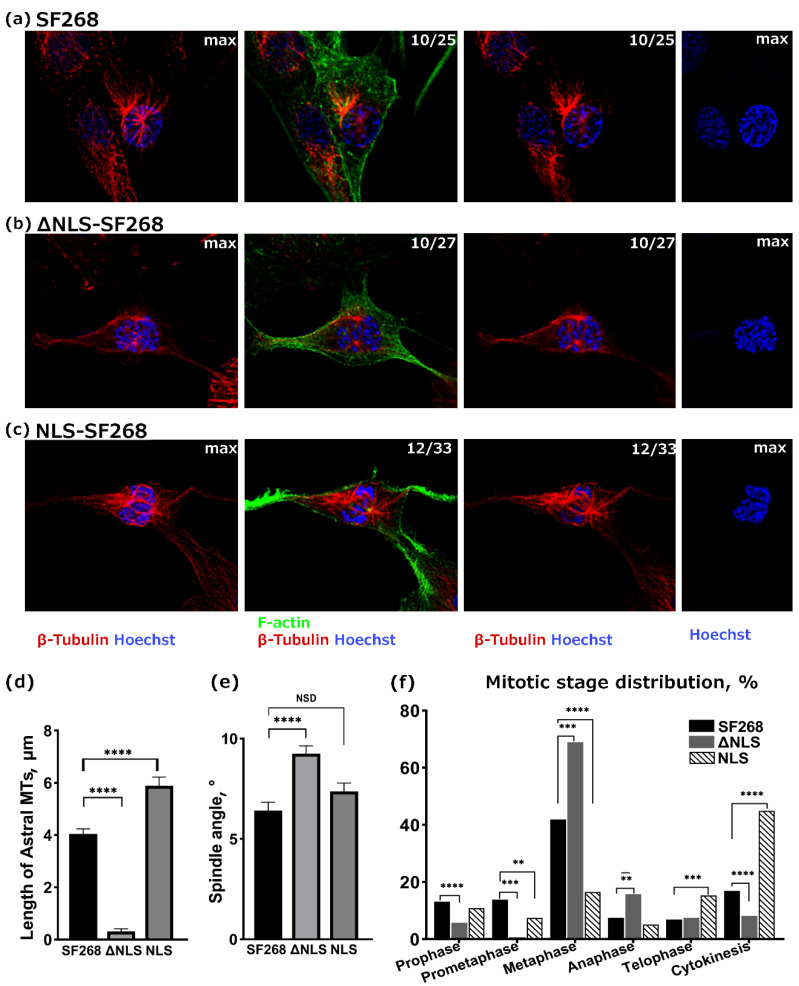
Aster formation is drastically and inversely regulated by NLS and ΔNLS neurofibromin isoforms. Synchronized cells were fixed with PFA, immunostained for β-tubulin (red) and co-stained with Hoechst for chromatin (blue) and with phalloidin for F-actin (green) visualization. Prophase images of (**a**) parental, (**b**) ΔNLS and (**c**) NLS SF268 cells were captured as confocal Z-sections of 0.34 μm steps. Left column of panels contains compilations of all Z-sections containing β-tubulin and chromatin signals (maximal intensity projection), i.e., a z-stack of 8.5 μm, 9.18 μm, and 11.22 μm, for a, b, and c respectively. Middle columns contain single sections at 3.40, 3.40, 4.08μm from the growing surface, corresponding to the indicated section number (over total sections), with (middle column) or without (right column) the co-projection of F-actin optical signals. Right panels are the maximal intensity projection of Hoechst staining. Quantifications of (**d**) astral length, (**e**) spindle angle, and (**f**) mitotic stage percentages. Bracketed bars in d and e represent the means and standard errors of measurements in 25–30 cells per cell line from each of 3 separate experiments with synchronized cells and in f, mitotic stage distribution of asynchronous cells (*n* = 800 mitotic figures for each cell line from five different experiments). *p* values, based on $\bar{x}$ ± SE, are: ** ≤ 0.01, *** ≤ 0.001, **** ≤ 0.0001; NSD, no significant difference.

We next sought to investigate whether the effects on centrosomes, imposed by the presence or absence of an NLS in the sequence of neurofibromin, also affected formation of mitotic asters, by co-immunostaining cells synchronized at prophase with β-tubulin and neurofibromin (Figure 6). More specifically, in parental SF268 glioblastoma cells and in cells expressing NLS-neurofibromin, asters clearly bore MTs extending towards the cell cortex (astral MTs) and inwards (polar, interpolar MTs), while in cells lacking NLS neurofibromin projections to the cortex were absent (Figure 6 left column, a and c versus b). This is best appreciated when only the one plane (of 0.34 μm, the limit of confocal imaging resolution for the 63x lens we used) that contained the maximum signal of β-tubulin is viewed (Figure 6, all middle and right columns of panels) and the prominently tangent projections of astral MTs become apparent in both parental and NLS-SF268 cells, as well as the almost complete loss of such astral MTs in ΔNLS-SF268 (Figure 6, a and c versus b).

Using the ImageJ plugin Single Neurite Tracer within an area defined as the outside half of a circle centered on a centrosome, the length of all visible microtubules that contacted the cortex was quantified in cells at metaphase. As expected from the spindles imaging (Figure 4 and Supplementary Figure S4), the clearly visible tuft of microtubules in SF268 had an average length of 4.0 ± 0.19 μm (Figure 6d), elongated astral MTs with an average length of 5.9 ± 0.33μm were steadily recorded for cells expressing NLS-neurofibromin (Figure 6d), and a robust loss in cells expressing ΔNLS neurofibromin was established (Supplementary Figure S4c, versus a and b), with a mere 5% of cells possessing only a few, short astral MTs if any (Figure 6d). Although incompletely understood, proper astral formation is required for spindle position and aberrations of astral MTs correlate well with spindle misorientation (e.g., [4]). Consequently, the abnormal patterns of astral MT projections with loss of NLS-neurofibromin led us to examine whether spindle positioning was also affected. Indeed, loss of astral MTs along with loss of NLS-neurofibromin led to differential positioning of the usually parallel to the growing cell surface spindle by several degrees (Figure 6e).

Abnormal positioning of the spindle typically associates with altered times spent at mitotic stages [6,71]. Thus, flow cytometry results showed that NLS transcript knockdown significantly increased the percentages of cells in G2/M phase: 15.2% with ΔNLS-expression, compared to 10.6% in parental and 9.8% with NLS-expression, while doubling time analysis showed that the 38 h cell cycle of parental SF268 (G1: 17.1 h, S 10.3 h, G2 6.8 h, M 3.8 h [18]) was now extended to 40.2 h in cells lacking NLS isoforms, but not affected in cells lacking ΔNLS neurofibromin (Supplementary Figure S5). Quantification of the mitotic stage distribution for each cell type revealed that loss of NLS-neurofibromin elicited a great increase in cells in metaphase (Figure 6f). Because of the aster formation abnormalities in ΔNLS cells (Figure 4), we scored cells with full length spindles and MTs curved towards the equator as metaphasic, and, as a result, negligible percentages were recorded for prometaphasic ΔNLS cells. The high percentage of metaphasic ΔNLS cells most likely reflected longer time spent at metaphase and correlates well with the increase in the overall duration of the cell cycle. In contrast, NLS cells had significantly lower percentages in pro- and metaphase over parentals, which, combined with higher percentages in cytokinesis, reflected an overall acceleration through metaphase. Considered together, these results document for the first time that neurofibromin actively participates in the formation of mitotic asters and the progression of mitosis. Moreover, these data further support the notion that NLS- and ΔNLS-neurofibromins may exert opposing effects during aster formation and spindle assembly, as, in parental cells, these two parameters appear to be the arithmetic sum of the results obtained with each isoform type.

### 3.4. Chromosome Segregation Fidelity Requires NLS-Neurofibromin Expression

**Figure 7:**
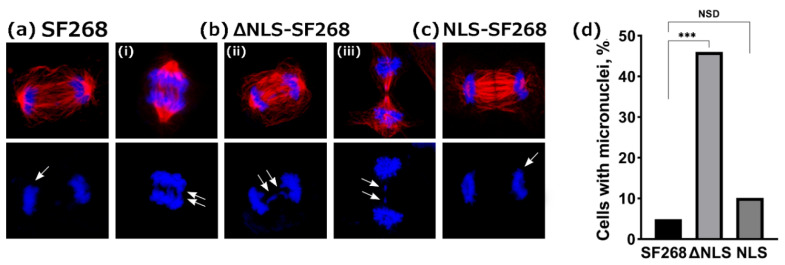
NLS-Neurofibromin is required for accurate chromosome segregation. Immunofluorescence confocal images of (**a**) SF268, (**b**) ΔNLS-SF268, and (**c**) NLS-268 synchronized cells, treated with DSP prior to fixation and stained for β-tubulin (red) and chromatin (blue). SF268 and NLS-SF268 spindles show proper chromosome segregation during late anaphase, whereas the anastral ΔNLS spindles in anaphase (i, early and ii, late) and cytokinesis (iii) show high frequency of segregation errors, highlighted by arrows. (**d**) Quantification of micronuclei, as described in Supplementary Figure S6, shows high frequency of micronuclei occurrence in ΔNLS- but not in NLS-SF268 cells. Bars are the percentage of cells with micronuclei from an *n* ≥ 500 from 3 different experiments and *p* value, based on $\bar{x}$ ± SE is: *** ≤ 0.001; NSD, no significant difference.

The metaphase phenotypes along with the anaphase time variability, observed with expression of ΔNLS-neurofibromins only, led us to investigate next chromosome segregation events during anaphase and telophase in fixed cells (Figure 7a–c). We found that, in parental and NLS-neurofibromin expressing cells, chromosomes appeared to move in a coordinated manner towards the opposed poles, while chromosome compaction was readily seen (Figure 7a,c, arrows). In cells expressing only ΔNLS-neurofibromins, these parameters were again inversely regulated, that is, despite the prolonged time spent at metaphase, there was a significant increase in cells with chromosome segregation errors (>40%), mainly lagging chromosomes (Figure 7, arrows in b.i and ii), and occasionally chromosome bridges, without much obvious compaction of the main chromosome mass. Similar delays in chromosome compaction in cells lacking NLS-neurofibromin were observed in telophase (e.g., Figure 7, arrows in b.iii). Tracking the effects of the described aster and spindle assembly perturbations, and of the chromosome segregation errors in similarly stained cells, we found that the missegregated chromosomes resulted in a high frequency of micronuclei formation, that is an almost 5-fold increase in the numbers of cells containing micronuclei within in 2 passages (P2) or ~10 mitotic cycles (Figure 7d and Supplementary Figure S6a–c). Taken together, these results strongly suggest that neurofibromin isoforms, changed only by the inclusion, or not, of an NLS sequence have opposing, possibly complementary when both expressed, functions, that are required for mitotic aster formation and spindle assembly for efficient chromosome congression and error correction, at least in an astrocytic cellular background.

## 4. Discussion

When we previously probed the nuclear functions of the tumor suppressor neurofibromin, we found that it actively accumulates, in a Ran-dependent manner, in the nucleus prior to mitosis to participate in proper chromosome congression [18], as do many other tumor suppressors (e.g., [14,15,16,17,19,20,21,72]). We now further elaborate on these findings with experimental evidence that supports a direct function of neurofibromin in mitotic aster and spindle assembly for proper chromosome congression and faithful genomic transmission.

Unsurprisingly for such a large gene and protein, functional neurofibromin isoforms have been reported, which are produced by alternative exon splicing in a cell type-specific mode. Splicing of exon 31, which generates neurofibromins with different RasGAP activities has been previously addressed [34,45], whereas the importance of the inclusion of the C-terminus NLS-bearing exon 51 [52] has not been previously explored. By using stable transfections with lentiviruses carrying shRNAs that specifically knockdown NLS- or ΔNLS-*NF1* transcripts in the human glioblastoma cell line SF268, we were able to produce glioblastoma cellular backgrounds that conditionally express either NLS- or ΔNLS-*NF1* transcripts (Supplementary Figure S1). In this manner, we were able to dissect the roles of NLS or ΔNLS neurofibromins, which differ in the inclusion of the corresponding to exon 51 sequence of 41 amino acids and the expression of a bipartite NLS sequence (Scheme 1).

Lack of nuclear neurofibromin immunoreactivity in the ΔNLS-SF268 cells (Figure 1 and Table 1) triangulates our previous results with Ran variants overexpression and nuclear subfractionation approaches, and further establishes that this large protein may only enter the nucleus through the Ran/importin system, that is only through its NLS [18,48]. A second requirement for its cell-cycle regulated nuclear entry is phosphorylation by Protein Kinase C (PKC) on serine 2808, a residue relatively close to the NLS (Scheme 1). This residue is retained in both isoforms, yet in silico analysis shows that three other potential phosphorylation sites within the 41 amino acid sequence encoded by exon 51 are lost in ΔNLS neurofibromins, namely a PKC site on T2519, a tyrosine kinase site on Y2556, and a site for Casein kinase 2 (CK2) on T2554. These phosphorylations may also play roles in the nuclear import of NLS neurofibromins in preparation of mitosis, especially the CK2 site, as this significant for cell cycle progression kinase [73] phosphorylates the yeast neurofibromin homologue Ira2 to prevent its degradation [74]. Considered together, our findings further establish the nuclear import of NLS neurofibromins and exclusion of ΔNLS ones; however, whether one functions dominantly over the other in the cytosolic area or after nuclear envelope breakdown is not directly addressed.

Unlike results with Western blotting, in immunoprecipitations with three antibodies against the N-terminus, different amounts of endogenously expressed neurofibromin were recovered from ΔNLS- or NLS-SF268 cell lysates (Supplementary Figure S1). Because protein conformation in cell lysates differs and may closer resemble endogenous states than those attained after fixation or exposure to SDS, we reasonably presume that inclusion, or not, of the 41 amino acids of exon 51 may indeed alter the conformation of the molecule. With an estimated molecular mass of ~320 kDa, neurofibromin has been repeatedly shown to migrate as a 220–250 kDa protein in SDS polyacrylamide gels [32,48,49,59,60,75], most likely due to the known intramolecular interactions between its domains and specific protein folding. At any case, the expected differential conformation of the ΔNLS or NLS neurofibromins would also impact its well established intermolecular interactions [18,35,60,75]. In support of this argument, colocalization with F-actin [18,51], an interaction carried out by the further upstream CSRD domain in the N-terminus [47,49], was differential for the two isoforms (Figure 1 and Table 1). Moreover, colocalization analyses with β- and γ-tubulins showed that both NLS and ΔNLS neurofibromin isoforms retain the ability to bind tubulins, although the affinity of NLS is higher for β- and lower for γ-tubulin when compared to ΔNLS (e.g., Figure 1; Table 1 and Table 2).

These differential effects of each neurofibromin type, collectively the effects on cytosolic MT intersection occurrence (Figure 2) and cytosolic or spindle β-tubulin polymer regulation (Table 1 and Figure 4e), strongly support the previously suggested role of neurofibromin as a MAP. Experimentally, at least three neurofibromin domains have been previously identified to bind tubulins, namely GRD, SEC14, and CTD. Affinities to tubulin for the first two domains were explored for regulation of neurofibromin’s RasGAP activity [33,34,36] and the third for baiting neurofibromin associated proteins [35] or for nuclear import studies [18]. More specifically for CTD, high affinity to GFP-CTD (+NLS) was identified for α-, β- and γ-tubulins [18], as well as for the Collapsin response mediator protein 2 (CRPM2) [35], a plus-end MAP [76] thoroughly studied for its abilities to promote MT assembly during neuronal axon formation and to endorse actin filament stability at growth cones [77]. Interestingly, CRPM2 also decorates the mitotic spindle and midzone structures, while its depletion leads to altered spindle position, destabilized astral MTs, and segregation errors [78]. Thus, we performed sequence comparisons (EMBOSS Water), which showed a 50% identity/similarity of the MAP domain of CRMP2 [78] with neurofibromin residues 1815–1839 (Supplementary Figure S7). At higher percentages of similarity, we found three additional small Tau-like motifs [79] containing the critical lysine for α-tubulin binding (Supplementary Figure S7), one of which corresponds to codons apposed to exons 50–51 or 50–52 junctions and are thus mostly likely to be affected.

Moreover, the direction of the ΔNLS or NLS knockdown effects was most often opposite, suggesting that the two possibly interact when both present in wild type cells. Indeed, neurofibromin has been shown recently to dimerize and affinity precipitation experiments have pinpointed CTD as the likely mediator [48]; whether this analysis included NLS or ΔNLS variants was not, however, disclosed. At any rate, the interesting question remains, if loss of the amino acids encoded by exon 51, suffice to produce a different MAP. Numerous examples exist where one to few amino acids may change the functional properties of a protein, by imposing changes on protein conformation and/or sites for post-translational modifications, and our data, starting with the immunodetection experiments, suggest that this is the case for the ΔNLS and NLS isoforms. By the same token, the expression of two closely related isoforms yet with differential effects on MT structures further suggests that an extra layer of regulation on MT dynamics is thereby served.

The differential properties of the NLS and ΔNLS isoforms are further highlighted by the effects on centrosome size, as assessed by their γ-tubulin immunopositive volume (Table 2 and Figure 5), and on MT nucleation prior to nuclear envelope breakdown, when the relatively sparse microtubule formations of interphase cells transfigure into a bipolar spindle. In certain species, cell types, or experimental set-ups that exemplify non-centrosomal MT nucleation, spindles are formed and mitoses are completed [80,81]. Yet, in most animal cells, the centrosome is the major MTOC for spindle MT assembly and duplicated centrosomes serve as poles for already nucleated astral MTs that radiate towards and interact with a core machinery, anchored on the cell cortex, to orient the spindle. Furthermore, astral poles nucleate highly dynamic MTs from the spindle [82], K-fibers from kinetochores [7,31,83], and interpolar bundles that elongate the spindle [4,5,6]. A large body of literature has postulated that γ-tubulin and its several associated proteins in higher eukaryotes form a large ring complex (γ-TuRC) that serves as the major template for α-tubulin subunits of MT minus ends [84]. Still, the control of MT nucleation remains incompletely understood, for example γ-TuRC [85] is dispensable for this purpose (e.g., [86]). This, in turn, underscores the role of MAP-dependent regulation of nucleation, which may increase, as does XMAP125 [87] often in synergy with structurally unrelated MAPs [88], or inhibit nucleation from centrosomes, as does stathmin [89]. While we may only speculate on how nucleation at the duplicated, larger centrosomes (Figure 5) appeared to be prevented for MTs radiating to the cell cortex, yet allowed for those from the chromosome area when only ΔNLS neurofibromins are expressed (Figure 6), it is feasible that ΔNLS neurofibromins may impede partitioning of tubulins from polymer to soluble dimer pools at mitotic entry [90], in a manner similar to the temporal mode of action of stathmin [89]. Another possibility is that the decreased affinity of ΔNLS neurofibromins to actin (Figure 1 and Table 1) may interfere with interactions of actin and MTs, as also demonstrated by the aberrant pattern of cell migration (Figure 3, Supplementary Figure S3, and Supplementary Videos S1–3). Indeed, actin and MT interactions at the nuclear envelope during G2 may have significant impact on centrosome positioning for cell division and spindle orientation [91,92]. Lastly, we may not exclude the possibility, which has yet to be addressed, that the RasGAP activities of NLS and ΔNLS isoforms vary and thus different transcriptional regulation on MT nucleation regulators is exerted via Ras-dependent pathways.

Astral, γ-TuRC nucleated MTs are essential for proper anchoring to the cell cortex and correct mitotic spindle orientation, a fundamental process in embryo development and in tissue renewal [93]. Appropriately, a number of diverse families of proteins across species have been shown to impact the timely assembly and dynamic maintenance of astral MTs, mostly through depletion studies. Among those, few studies record shorter or weaker astral MTs, while the astral loss, to the extend we report here with NLS-neurofibromin depletion and expression of only ΔNLS isoforms (Figure 4 and Figure 6), has not been reported. The closest parallelism to the effects of NLS-neurofibromin knockdown, namely increased cytosolic tubulin polymer (Table 1) with fewer MT intersections (Figure 2), loss of astral MTs, delay at metaphase and abnormal chromosome congression (Figure 4 and Figure 6), and finally abnormal chromosome segregation and increased micronuclei frequency (Figure 7), is with ablation of EB2, an important MT plus-end tracking MAP. Unlike EB1 and EB3, EB2 preferentially binds to microtubule lattices [94], in a phosphorylation-dependent manner to protect MT dynamic instability during mitosis [95]. Ablation of EB2 produces straighter, bundled cytosolic MTs with fewer MT intersection events [96], while its functional ablation (phosphoablating mutants) leads to a marked delay in anaphase onset, and abnormalities in chromosome congression and genomic transmission [95]. Similar depletion phenotypes have suggested that the multidomain, multifunction adaptor proteins ALG-2-interacting protein X (ALIX) [97] and the Receptor of Activated C Kinase 1 (RACK1) [98] are also essential for proper astral MT elongation, spindle orientation and chromosome segregation, acting through regulation of motor protein (RACK1) or other MAPs (ALIX); these possibilities are worth pursuing experimentally for NLS neurofibromin. In contrast, most parameters assessed with ΔNLS-depletion and expression of NLS neurofibromin diverge only slightly from those registered for the parental SF268 cells, namely longer astral MTs and shorter spindles (Figure 4 and Figure 6). Such mild effects have been reported after silencing motors from the kinesin family of MAPs, specifically MCAK [99] and kinesin-8 (Kif18b) [71,100], and were interpreted as evidence for preferential actions on specific MT subpopulations. Overall, the data we obtained, when assessing a number of parameters, point out that the shorter spindles in NLS cells were more efficient than their ΔNLS counterparts in protecting the genome and ensuring faithful chromosome segregation.

Expression of ΔNLS neurofibromins did not appear to impede MT nucleation as severely at the centrosome/pole beyond prophase, and we may deduct that mitotic factors, already accumulated in the chromosome area, drastically enhanced nucleation; then kinetochore-derived microtubules interacted laterally with pole microtubules (Figure 6) for kinetochore loading [101]. It is also possible that the augmin-mediated, centrosome-independent nucleation from preexisting MTs within the spindle [81,102,103] was upregulated, if the dramatic spindle geometry of some prominent thick MT formations with queued up chromosomes is to be interpreted (Figure 4). As the spindle equator area was the most devoid of tubulin signals, we may assume that non kinetochore, bridging MTs [104] were also sparse and late to develop. The patterns we observed strongly resemble the “prometaphase ring or belt” in the human diploid cell line RPE1, caused by unstable lateral interactions between kinetochores and microtubules that dominate early prometaphase [70]. Computational modeling predicts that such a toroidal distribution would enhance exposure of kinetochores to a high density of MTs and facilitate amphitelic attachments [70]. As we very rarely, <0.01%, observed this configuration in parental or NLS-SF268 cells, we assume that they must execute chromosome prepositioning extremely fast, whereas ΔNLS cells were arrested at this stage for a long time, or till most defects in spindle assembly were corrected and stable end-on attachments became possible. Indeed, cells were able to undergo mitosis, and segregation abnormalities, mostly lagging or unattached chromosomes (Figure 7) at anaphase, were less drastic than those expected from the dramatic spindle and chromosome congression geometries. Despite, however, this remarkable correction, the frequency of micronuclei was significantly increased (Figure 7 and Supplementary Figure S6). Moreover, this defect accounted as the only irreversible event upon doxycycline removal. Observed at elevated frequencies in cancer cell lines, micronuclei frequency is a predictive biomarker for genetic instability and for rapid karyotype evolution [105], as their few chromosomes, unprotected from DNA damage, may undergo chromothripsis and chromoanasynthesis [106] and then get incorporated into the genome of the host cell within just 1–2 mitoses [107].

Summarizing, our studies strongly demonstrate that inclusion, or not, of the amino acids encoded by exon 51 may alter the presentation of neurofibromin’s microtubule-binding domains, which, in conjunction with the ability or not to enter the nucleus at late S, may explain the role of the molecule in spindle assembly and chromosome segregation. Finally, when the observed phenotypes are considered together, our hypothesis that NLS and ΔNLS neurofibromin isoforms may balance each other out for the regulation of astral MT and spindle dynamics is further strengthened.

## Figures and Tables

**Table 1:** Quantitative intensity and colocalization analysis.

	Nucleus	Cytosolic Area				Spindle
	Neurofibromin Intensity	Colocalization, F-Actin and Neurofibromin		Colocalization, β-Tubulin and Neurofibromin	β-Tubulin Intensity	Colocalization, β-Tubulin and Neurofibromin
		All	Lamellipodia			
SF268	9.80 ± 1.16	0.17 ± 0.01	0.41 ± 0.07	0.23 ± 0.02	28.83 ± 0.47	0.46 ± 0.04
ΔNLS-SF268	2.21 ± 0.30 ***↓	0.07 ± 0.04 *↓	0.16 ± 0.05 ****↓	0.21 ± 0.02 †	73.75 ± 3.06 ****↑	0.52 ± 0.02 †
NLS-SF268	9.32 ± 0.80 †	0.26 ± 0.01 *↑	0.55 ± 0.04 *↑	0.33 ± 0.01 ****↑ ^1^	25.01 ± 0.19 †	0.66 ± 0.02 ***↑

Colocalization of β-tubulin or F-actin and neurofibromin, was measured using Volocity and Pearson’s Correlation Coefficient (PCC) on confocal images; the area of the nucleus was defined by the Hoechst staining signals. Nuclear neurofibromin intensity and the intensity of β-tubulin in the cytosolic area were measured using IMARIS on confocal images; numerical values represent the mean intensity per cell area in arbitrary units ± SE from 40 randomly chosen cells, from each of 5 experiments. *p* values refer to statistical differences between each genetically modified cell line and the parental SF268 cells, and arrows indicate the direction of these differences. ^1^ the statistical difference between ΔNLS-SF268 and NLS-SF268; *, *p* < 0.05; ***, *p* < 0.001; ****, *p* < 0.0001; †, no significant difference.

**Table 2:** Quantitative intensity of γ-tubulin and neurofibromin isoform colocalization analysis in cells modified for expression of *NF1* transcripts.

	Centrosome		
	γ-Tubulin Volume, μm^3^	γ-Tubulin Intensity	Colocalization, γ-Tubulin and Neurofibromin
SF268	2.85 ± 0.14	58.7 ± 4.61	0.68 ± 0.02
ΔNLS-SF268	5.35 ± 0.31 ** ↑	67.5 ± 2.93 †	0.62 ± 0.03 †
NLS-SF268	2.87 ± 0.39 †	62.6 ± 0.88 †	0.48 ± 0.06 ** ↓

Colocalization analysis using Volocity^®^ and Pearson’s correlation coefficient (PCC) of γ-tubulin and neurofibromin. IMARIS software was used to calculate the centrosome volume and the intensity of γ-tubulin. Numerical values represent the mean ± SE in arbitrary units and *p* values refer to the statistical differences from comparisons between each genetically modified cell line and the parental SF268 cells (20 cells per cell line from each of the 5 experiments); arrows indicate the direction of these differences; **, *p* < 0.01; †, no significant difference.

## Supplementary Materials

The following are available online at https://www.mdpi.com/2073-4409/9/11/2348/s1, Figure S1: Validation of knockdown efficiency using shRNAs against ΔNLS- and NLS-NF1 mRNA transcripts by qPCR and Western Blotting, Figure S2: Single planes of β-tubulin or F-actin co-localization with neurofibromin, Figure S3: Wound healing assay, Figure S4: Astral MT networks after fluorescence signal enhancement, Figure S5: Loss of NLS-neurofibromins elicits increases in the percentage of cells in G2/M phase and in doubling time, Figure S6: Knockdown of NLS neurofibromin elicits high frequency of micronuclei, Figure S7: Tubulin binding motifs of neurofibromin, Video S1: Time-lapse video microscopy of SF268 cells, Video S2: Time-lapse video microscopy of ΔNLS-SF268 cells, Video S3: Time-lapse video microscopy of NLS-SF268 cells.
