# Supplementary material for: Nuclear Isoforms of Neurofibromin Are Required for Proper Spindle Organization and Chromosome Segregation
Source: Cells. 2020 Oct 23;9(11):2348. doi: 10.3390/cells9112348 (PMC7690890; doi:10.3390/cells9112348)
Supplement: Supplementary file 1 [file cells-09-02348-s001.zip › cells-887858-supplementary-for publication/cells-887858-supplementary-for publication.pdf]

Supplementary Material

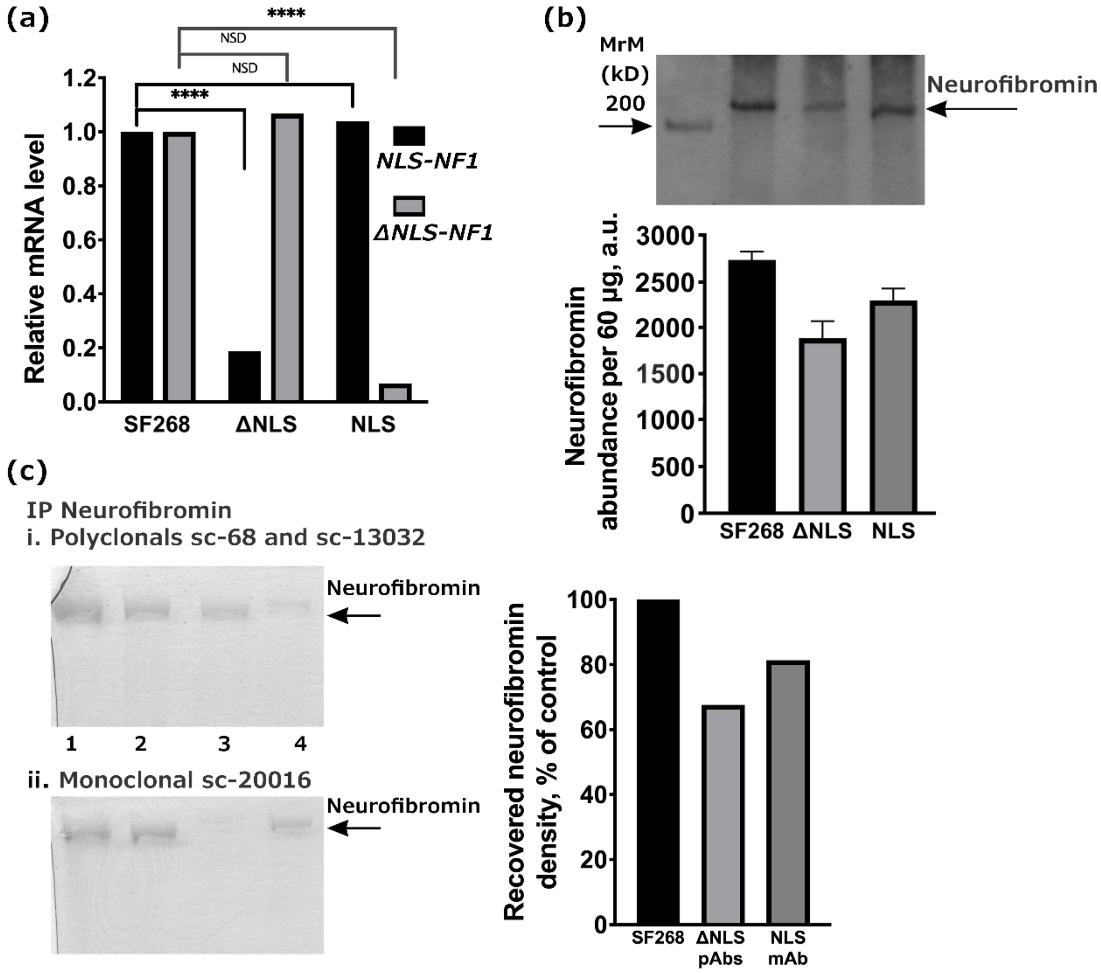

Supplementary Figure 1. Validation of knockdown efficiency using shRNAs against ΔNLS- and NLS-*NF1* mRNA transcripts by qPCR and Western Blotting. (**a**) Quantitative analysis of qPCR results shows effective knockdown of endogenous ΔNLS- or NLS-NF1 transcripts using the chosen shRNAs. *NF1* transcript levels were analyzed using primers designed to amplify either NLS or ΔNLS isoforms, as described in Materials and Methods. Experiments were performed in triplicates and data are presented as percentages of their normal control (SF268 cells). P values were calculated by Mann–Whitney U-test, $n = 3$); ****, $p < 0.0001$; NSD, no significant difference. (**b**) Quantitative analysis of neurofibromin protein expression in ΔNLS- and NLS-SF268 cells confirms effective shRNA-dependent knockdown of corresponding transcripts. A representative image of Western blot analysis, performed as described in Materials and Methods, is shown in the upper panel; left arrows point to the 200 kDa pre-stained marker (sc-2361, Santa Cruz). Bar graph in the lower panel shows the results from the densitometry analysis of Western blot scans and postulates a 30% reduction of neurofibromin abundance in ΔNLS-SF268 and ~20% in NLS-SF268. Data (columns) are the mean values ± SEM values from 7 independent experiments. (**c**) Differential recognition of endogenous neurofibromin by antibodies raised against N-terminus epitopes indicates dissimilar conformations of ΔNLS- and NLS-neurofibromins. Left panels contain representative images of silver stained gels, containing immunoprecipitated neurofibromins after SDS–PAGE (7% polyacrylamide). Immunoprecipitations were performed as described previously (e.g., [18,49]). Briefly, SF268 naïve (lanes 1), SF268 stably expressing mock shRNA (lanes 2), ΔNLS-SF268 (lanes 3), or NLS-SF268 (lanes 4) were lysed in RIPA buffer containing protease and phosphatase inhibitors, the insoluble material was removed by centrifugation, and the resulting supernatants were assayed for protein content with

the DC Biorad kit (Biorad, Hercules, CA, USA). Equal amounts of lysates were then precleared by incubation with normal rabbit or mouse IgGs conjugated to protein A/G-agarose beads (Santa Cruz) and these supernatants were incubated with (i) combination of two rabbit polyclonal antibodies (pAbs), sc-68 and sc-13032 or (ii) a mouse monoclonal (mAb), sc-20016, all from Santa Cruz; immunoprecipitates were analyzed by SDS–PAGE (7% polyacrylamide). Bar graph in the right shows the densitometry analysis (Image J) results from several experiments, presented as percentages of the control (naïve SF268). Values for ΔNLS-SF268 were calculated from immunoprecipitation densities recovered with pAbs (e.g., c-i, lane 3), and for NLS-SF268 from densities recovered with the mAb (e.g., c-ii, lane 4). The results closely resemble those obtained with Western blot analysis (shown in b). We are interpreting the immunoprecipitation results as indicating altered conformations of the ΔNLS and NLS neurofibromins that are recognized by these antibodies; this possibility should be further explored experimentally as a potential tool for several purposes.

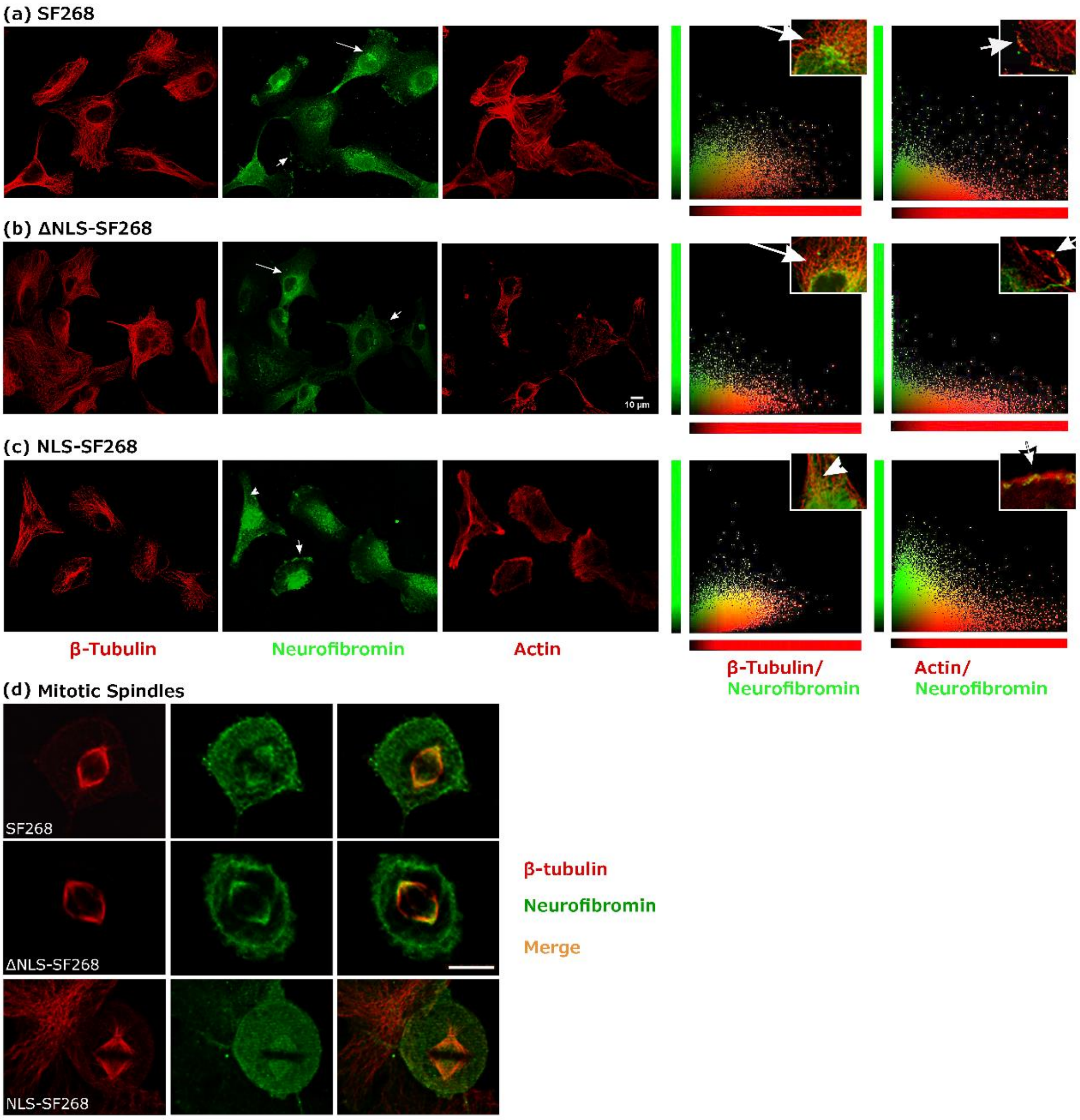

Supplementary Figure 2. Single planes of β-tubulin or F-actin co-localization with neurofibromin. Panels in columns 1–3 show representative single focal planes (0.34 µm) of the confocal stacks shown β-tubulin (red) and neurofibromin (green); co-staining for F-actin is also presented in red for easier viewing of signal colocalization. Scatter plots in the last two columns show signal intensity of each voxel in the plane, as indicated in the labels; insets show regions, denoted with arrows and detailedly described in Results for Figure 1, at 300% magnification. (**d**) Rows contain representative single focal planes (0.34 µm) of the confocal stacks shown in the last column of Figure 1: β-tubulin (red), neurofibromin (green), and then merging of the two; bar = 10 µm.

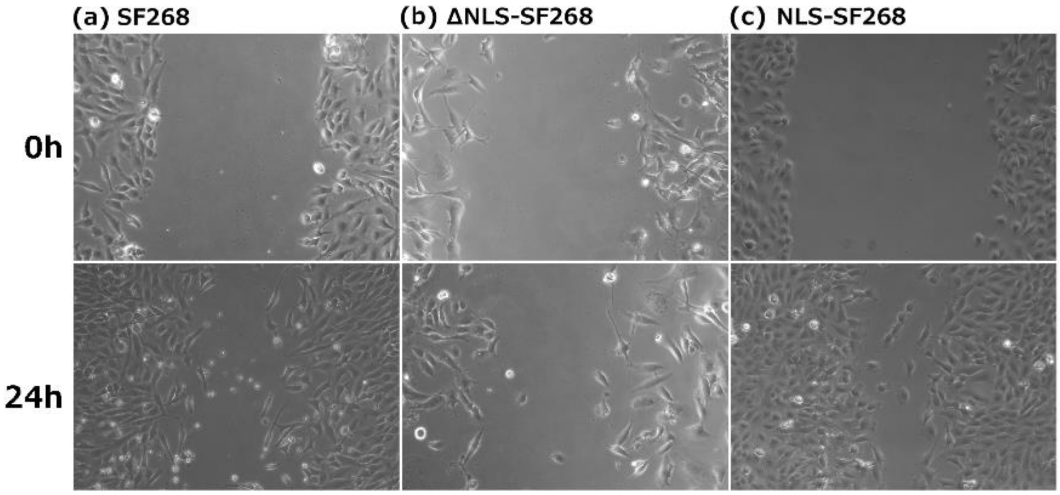

Supplementary Figure 3. Wound healing assay. (**a**) SF268 (**b**) ΔNLS and (**c**) NLS cells were scratched and photographed in several areas under phase in a Zeiss 200M inverted microscope (upper panel); coordinates of these areas were marked with the appropriate stage tool and the same areas were photographed again after 24 h (lower panel). Wound area coverage was assessed as described in Methods. Bar = 10 μm in all panels.

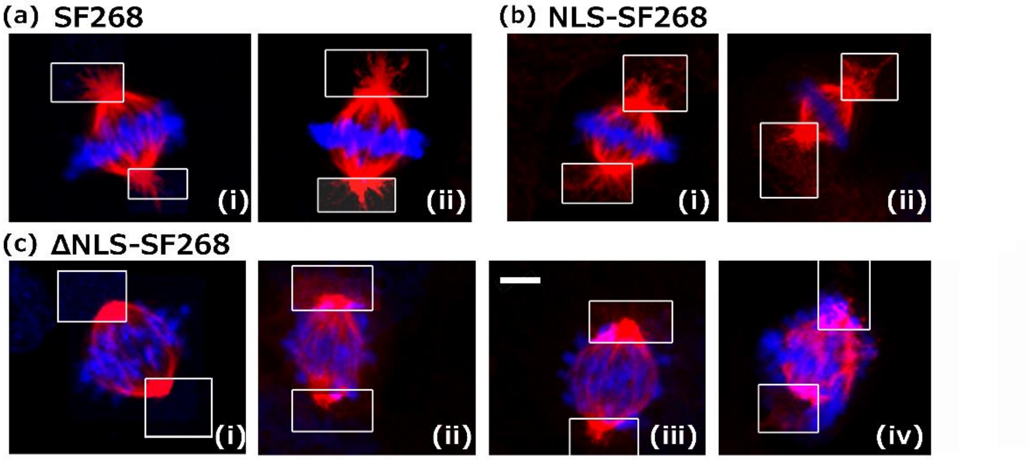

Supplementary Figure 4. Astral MT networks after fluorescence signal enhancement. Panels are identical to those presented in Figure 4 a–c, albeit fluorescence signal in the indicated areas (white outlines) has been enhanced manually by 3-fold to highlight astral MT patterns (confocal images were acquired at levels that the rest of the spindle was not saturated). This enhancement highlights the lack of astral MTs tufts in ΔNLS-SF268 cells.

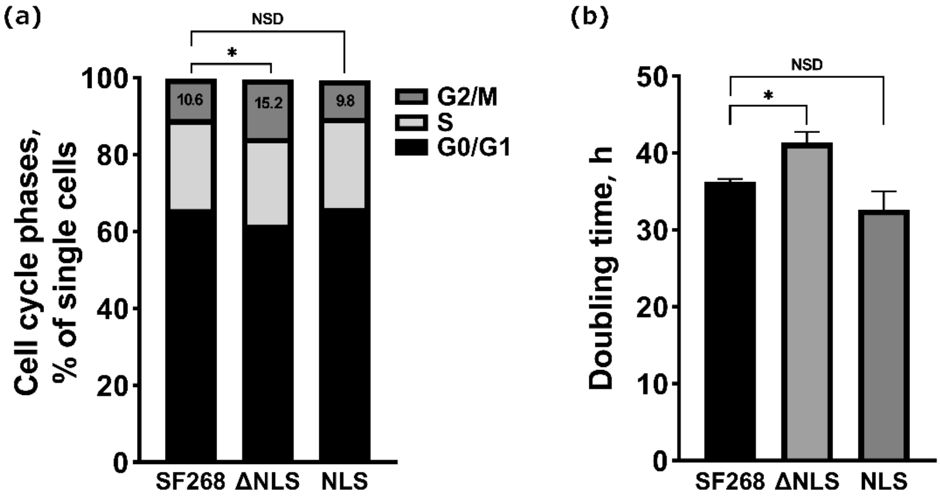

Supplementary Figure 5. Loss of NLS-neurofibromins elicits increases in the percentage of cells in G2/M phase and in doubling time. (**a**) Cell cycle analysis using propidium iodide (PI) staining and flow cytometry, was performed as described [18]. Briefly, asynchronous SF268, ΔNLS-SF268, and NLS-SF268 cells, plated at 10^4/cm^2 and cultured for 36 h, were fixed in ice-cold ethanol (70%) and stored at −20 °C (12–24 h); cells were then incubated with RNase A (Sigma; 0.1 mg/mL × 30 min), stained with PI (Sigma; 50 µg/mL × 1 h), and passed through a BD FACSCelesta™ sorter (FACS Facility, BRFAA). PI intensity was used to determine DNA content and identify G0/G1, S-phase, and G2/M populations. Stacked column graph depicts percentages of SF268, ΔNLS-SF268, and NLS-SF268 cells in G0/G1, S, or G2/M phase; indicated levels of statistical significance between frequencies in G2/M were calculated using the chi-squared test. (**b**) Bracketed bars represent the means and standard errors of doubling times in sister cultures, calculated using the equation Doubling Time = duration∗log(2)/(log(Final cell concentration)−log(Initial cell concentration)) [57]; numerical data were analyzed by ANOVA. All experiments were performed 5–7 times; * $p < 0.05$, NSD, no significant difference.

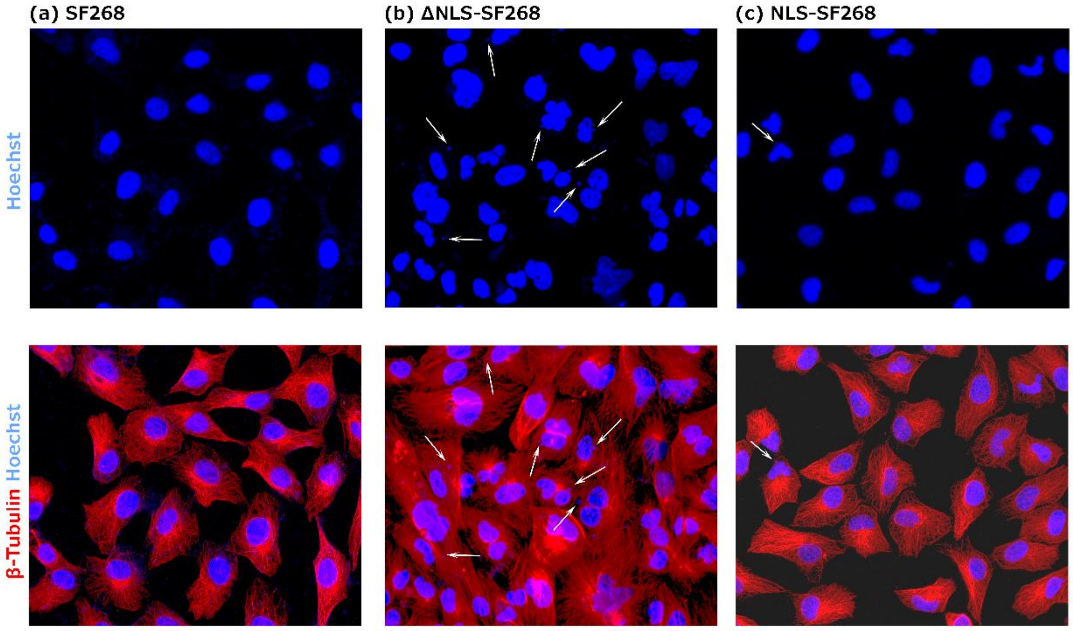

Supplementary Figure 6. Knockdown of NLS neurofibromin elicits high frequency of micronuclei. Confocal imaging of **(a)** Parental SF268, **(b)** ΔNLS-SF268, and **(c)** NLS-SF268 cells, fixed (DSP + PFA) and stained for chromatin with Hoechst 33258 (upper row) and β-tubulin (lower row, merge with Hoechst signals for orientation purposes), reveals high frequency of micronuclei with loss of NLS

neurofibromin (arrows in b versus a and c columns). Images are the maximum intensity projections of confocal serial 0.34 μm planes, containing all Hoechst signals. Quantitative scoring of micronuclei (graph in Figure 7d) was done by two independent, blinded observers on the same confocal or deconvolved images of randomly chosen fields from asynchronized cultures, using the ImageJ and Slidebook software, respectively. Scoring was performed on the same images, using the following criteria: micronuclei should be roundish or oval, the micronuclear boundary should be close to but clearly distinguishable from the nuclear boundary, and the micronuclei staining intensity should be at levels similar to those of the main nuclei.

(a) Neurofibromin (NF) and CRMP2

```
NF    1815 DSIPQHTKIRPKDVPGTLLNIALL 1839
           |:||:.|..|....||...||..|
CRMP2 549 DNIPRRTTQRIVAPPGGRANITSL 572
```

Identity: 41.7%
Similarity: 50%

(b) Neurofibromin (NF), Tau, and MAP2

1. NF 258 ESTKRK 263

```
         |:.|.|
TAU  380 ENAKAK 385
```

Identity: 50%

2. NF 815 RRMSHVSGGGSIDLSDTDSL 834

```
          |.:|:||..||||:.|:..|
TAU   406 RHLSNVSSTGSIDMVDSPQL 425
          ||:|:||..|||:|.::..|
MAP2 1788 RRLSNVSSSGSINLLESPQL 1807
```

Identity: 50%
Identity: 50%

3. NF 1010 QIKTK 1014

```
         .:|:|
TAU  255 NVKSK 259
```

Identity: 40%

4. NF 2494 SPRARKSM 2501

```
         ||.:.||.
TAU  235 SPSSAKSR 242
```

Identity: 50%

Supplementary Figure 7. Tubulin binding motifs of neurofibromin. Alignments, using the EMBOSS Needle database tools, were performed for sequence identity and similarity of neurofibromin and the MAP domain of (**a**) CRMP2 [78], and (**b**) Tau. Four small Tau motifs [79] were identified in neurofibromin, bearing the Lys residue responsible for cross-linking to α-tubulin, in: 1. the N-terminus; 2. the CSRD (previously identified by [32]); 3. right after CSRD; and 4. the CTD, corresponding to codons adjacent to the differentially spliced exon 51. All numbering of amino acids here and in the text correspond to GRDI-NLS neurofibromin of 2818aa (Ensemble transcript NF201).

Supplementary Videos 1–3. For time-lapse video microscopy, cells from each genetic background (1. SF268; 2. ΔNLS-SF268; 3. NLS-SF268), plated on 35mm glass bottom dishes (MatTek), were left to grow into a monolayer. The monolayer was then scratched, as described in Methods, and after 14 h was rinsed and bathed in Leibovitz's L15 (no phenol red). Phase images were captured every 2 min for a total of 4 h with an inverted Zeiss Axiovert 200M microscope equipped with a temperature controlled chamber; Slidebook features automatic generation of videos and macros to follow the movement of individual cells.
